# Beyond Ca^2+^ signalling: the role of TRPV3 in the transport of NH_4_^+^

**DOI:** 10.1007/s00424-021-02616-0

**Published:** 2021-10-19

**Authors:** Hendrik Liebe, Franziska Liebe, Gerhard Sponder, Sarah Hedtrich, Friederike Stumpff

**Affiliations:** 1 Institute of Veterinary Physiology, Freie Universität Berlin, Oertzenweg 19b, 14163 Berlin, Germany; 2 Department of Biology, Chemistry, and Pharmacy, Freie Universität Berlin, Oertzenweg 19b, 14163 Berlin, Germany; 3 Faculty of Pharmaceutical Sciences, University of British Columbia, Vancouver, Canada

**Keywords:** TRPV3, Ammonia, NH_4_^+^, Olmsted syndrome, Colon, Skin

## Abstract

Mutations of TRPV3 lead to severe dermal hyperkeratosis in Olmsted syndrome, but whether the mutants are trafficked to the cell membrane or not is controversial. Even less is known about TRPV3 function in intestinal epithelia, although research on ruminants and pigs suggests an involvement in the uptake of NH_4_^+^. It was the purpose of this study to measure the permeability of the human homologue (hTRPV3) to NH_4_^+^, to localize hTRPV3 in human skin equivalents, and to investigate trafficking of the Olmsted mutant G573S. Immunoblotting and immunostaining verified the successful expression of hTRPV3 in HEK-293 cells and *Xenopus* oocytes with trafficking to the cell membrane. Human skin equivalents showed distinct staining of the apical membrane of the top layer of keratinocytes with cytosolic staining in the middle layers. Experiments with pH-sensitive microelectrodes on *Xenopus* oocytes demonstrated that acidification by NH_4_^+^ was significantly greater when hTRPV3 was expressed. Single-channel measurements showed larger conductances in overexpressing *Xenopus* oocytes than in controls. In whole-cell experiments on HEK-293 cells, both enantiomers of menthol stimulated influx of NH_4_^+^ in hTRPV3 expressing cells, but not in controls. Expression of the mutant G573S greatly reduced cell viability with partial rescue via ruthenium red. Immunofluorescence confirmed cytosolic expression, with membrane staining observed in a very small number of cells. We suggest that expression of TRPV3 by epithelia may have implications not just for Ca^2+^ signalling, but also for nitrogen metabolism. Models suggesting how influx of NH_4_^+^ via TRPV3 might stimulate skin cornification or intestinal NH_4_^+^ transport are discussed.

**Supplementary Information:**

The online version contains supplementary material available at 10.1007/s00424-021-02616-0.

## Introduction

The multiple functions of channels of the transient receptor potential (TRP) family in general, and that of TRPV3 in particular, continue to be poorly understood [52]. The first TRP channel was cloned from a *Drosophila* fly mutant with visual impairment, resulting from a mutation that led to transient receptor potentials instead of the normal sustained response [47]. Since this time, 28 mammalian members of the family have been identified that form hetero- or homotetrameric assemblies and function as cation channels. Possibly owing to the initial discovery in the visual system of *Drosophila*, the family was initially associated exclusively with sensory perception. However, many members of the TRP channel family are highly expressed by non-sensory organs such as epithelial cells of the skin or the intestine [46, 52, 56, 74, 75], raising the question if sensory signalling is the end of the story.

From the first cloning of TRPV3 almost 20 years ago [57, 58, 71, 84], the high expression of TRPV3 in keratinocytes was noted in conjunction with a complete lack of expression in associated sensory dorsal root ganglion neurons. The channel is notoriously promiscuous with a low selectivity for Ca^2+^ [58]. Since TRPV3 is activated by warm temperatures above 33 °C, its primary function was initially thought to be in thermosensation, involving signalling via ATP or other molecules [45]. However, mice with a knockout of TRPV3 exhibited no obvious alterations in thermal preference behaviour [34] and instead, displayed a phenotype with curly hair and whiskers [17]. In mice, a gain of function mutation of TRPV3 caused a hairless phenotype with itchy skin, while in humans with Olmsted syndrome (OS), gain of function mutants cause severe palmoplantar hyperkeratosis [22, 40].

Despite progress, it remains uncertain why a gain of function of TRPV3 leads to hyperkeratosis in humans [22]. The skin consists of several layers of cells that differentiate as they grow upwards from the stratum basale, forming the stratum spinosum, the stratum granulosum, and the stratum corneum. This final layer consists of terminally differentiated keratinocytes or corneocytes, the cytosol of which is surrounded by cross-linked proteins. In the epidermal skin, this protein envelope is tightly linked to a further envelope of complexly organized lipids that seal the intercellular space. The corneocytes have lost cytoplasmic organelles and cell nuclei, but are important for skin hydration and reportedly continue to participate in signalling cascades such as cytokine-mediated initiation of inflammation [48]. Cells in the lower parts of the stratum corneum are tightly adjoined by corneodesmosomes, which are the main intercellular adhesive structures found in this layer. In an attempt to explain the hyperkeratosis found in Olmsted syndrome, a simple hypothesis suggests that a higher influx of Ca^2+^ through mutant TRPV3 leads to apoptosis [24]. However, skin peeling formulations that induce apoptosis cause exfoliation with a loss of corneocytes, the precise opposite of what is observed in OS [14, 52]. Hyperkeratosis occurs when the enzymatic disruption of the corneodesmosomes does not keep pace with the speed at which new corneocytes are formed [23].

The therapeutic success of targeting the epidermal growth factor receptor (EGFR) in OS [27, 86] supports a model in which EGFR stimulates keratinocyte differentiation into corneocytes. According to this concept, EGFR increases the activity of TRPV3, leading to influx of Ca^2+^ from the extracellular space into the stratum granulosum. Ca^2+^ activates transglutaminases [17, 23], which catalyse the cross-linking of glutamine and lysine residues of involucrin and loricrin, a key reaction in the formation of the highly resistant protein envelope. Synthesis of involucrin, loricrin, and a third important corneocyte protein, filaggrin, requires glutamine, which is produced intracellularly from NH_4_^+^ and glutamate via glutamine synthetase. A steady supply of ammonia is required both as a substrate and to activate glutamine synthetase [2, 19].

The model outlined above has been challenged by a study [85] of four separate TRPV3 mutants associated with the OS phenotype, including the most prominent point mutation glycine-573-serine (G573S) [22]. In that study [85], it was proposed that mutation of the TRPV3 channel interferes with the trafficking of the protein to the cellular membrane. This would prevent influx of Ca^2+^ into the cell, rather than enhancing it. Furthermore, it has been suggested that Ca^2+^ does not enter from the extracellular space but is released from intracellular stores after activation of calcium-sensing receptors [37]. It is also not quite clear why a non-selective cation channel such as TRPV3 is involved when the skin expresses a number of channels with a much higher selectivity to Ca^2+^, such as Orai1, TRPV6, or TRPA1 [37, 55]. The question arises if the low selectivity of TRPV3 might possibly also play a role in keratinocyte differentiation—for instance, by providing NH_4_^+^ for the production of the glutamine required for synthesis of involucrin, loricrin, and filaggrin as building blocks of the corneocyte envelope.

Apart from the skin, TRPV3 is also highly expressed by the apical membrane of enterocytes in intestinal epithelia such as the colon and the caecum, suggesting a role in the apical uptake of cations [46, 75]. A role in inflammatory signalling has been postulated, but attempts to correlate clinical findings in ulcerative colitis with expression of TRPV3 have been inconclusive [63]. A primary function in the absorption of Ca^2+^ can be ruled out since this clearly occurs in the small intestine via the highly selective Ca^2+^ channels TRPV5 and TRPV6, although transport of Ca^2+^ is possible [43]. However, the colon and the caecum absorb large quantities of ammonia [2]. Since NH_3_ is a highly polar molecule, it cannot simply diffuse through the lipid bilayer. Instead, it has been suggested that transport of ammonia (NH_3_) is facilitated by transport proteins such as the Rhesus-like glycoproteins or certain types of aquaporins [26, 30, 51]. Furthermore, functional studies have shown that both the colon and the caecum express an electrogenic pathway for the uptake of NH_4_^+^ via divalent-sensitive, non-selective cation conductances with permeability to NH_4_^+^ [46, 70], correlating with the apical expression of TRPV3 by both tissues [46, 75].

Our own interest in TRPV3 was sparked when looking for the pathway mediating the uptake of NH_4_^+^ from the rumen of cattle [39, 66, 69]. Having evolved from the oesophagus, the ruminal epithelium is a stratified squamous epithelium that, like the skin of many amphibians, has transporting properties [1, 8, 76]. In what is known as nitrogen recycling, urea is secreted from blood into the lumen, where microbes break up urea into ammonia which is then protonated and reabsorbed [31, 62, 72]. The same mechanism is found in the colon and caecum of monogastric species such as humans [72]. Since mammalian enzymes cannot break down urea, secretion of this waste product into a space colonized by microbes allows the host to reclaim the nitrogen contained in urea for the synthesis of glutamine and other non-essential amino acids in situations where dietary protein intake is low.

Intriguingly, the skin also secretes large quantities of urea, mostly via sweat [2, 5, 83]. While high concentrations of urea have keratolytic properties that are used to treat hyperkeratosis, the low concentrations (~ 7%) contained in sweat or in cosmetic products are generally considered to enhance skin hydration and barrier function via pathways that are incompletely understood [15]. Since many dermal bacteria express ureases [68], ammonia is released in large quantities, exceeding those in breath [38, 77]. It would certainly enhance evolutionary survival if at least part of this ammonia could be reabsorbed—e.g. via TRPV3—and utilized by dermal glutamine synthetase to produce glutamine as a precursor of the proteins required for keratin synthesis. In line with this hypothesis, inhibition of dermal urea transport inhibits production of transglutaminase, involucrin, loricrin, and filaggrin as proteins central to the process of cornification, although down-regulation of gene activity has been suggested as the mode of action [28].

The first purpose of the current study was therefore to investigate whether the human homologue of TRPV3 can conduct NH_4_^+^, as we have previously shown for the bovine homologue [39, 69]. A further purpose was to localize TRPV3 in a human skin equivalent [42] and, finally, to investigate the trafficking of the G573S mutant [85].

## Materials and methods

### Cloning of hTRPV3

Cloning was essentially performed as previously described [39, 69]. The human sequence of *TRPV3* (*hTRPV3*, NM_001258205.1) was obtained from Thermo Fisher Scientific GENEART (Regensburg, Germany) and tagged with a Hemagglutinin (HA) and a streptavidin (Strep) tag. The dual tag was placed at the N-terminus to prevent possible interference with a C-terminal PDZ binding motif found in some TRP-channels [60].

A number of experiments were performed using *Xenopus laevis* oocytes (referred to as *X*. oocytes or simply oocytes in the following, see below). For transfection, the HA-Strep-*hTRPV3* construct was subcloned into pGEM-HE-MCS (kindly donated by Prof. Blanche Schwappach, Georg-August-Universität, Göttingen, Germany) using the restriction sites HindIII and XbaI. The vector was linearized with the restriction enzyme MluI. RiboMAX Large Scale RNA Production System-T7 (Promega, Mannheim, Germany) was used for in vitro transcription to cRNA according to the manufacturer’s instructions.

For transfection of HEK-293 cells, the HA-Strep-*hTRPV3* construct was subcloned into pIRES2-AcGFP1 (Takara BioEurope, Saint-Germain-en-Laye, France) using the restriction sites NheI and XhoI. In the vector arrangement, the hTRPV3 coding sequence lies upstream, followed by an IRES sequence and, finally, the green fluorescent protein (GFP) coding sequence. Accordingly, hTRPV3 and GFP were separately expressed. The same method was utilized to construct a vector using a mutated sequence of the *hTRPV3* gene (Thermo Fisher Scientific GENEART) containing the point mutation G573S.

### Harvesting and injection of Xenopus laevis oocytes

*X.* oocytes were harvested and processed from the same frogs as in Liebe et al. [39] (supplement part A, permit G0025/16). Xenopus laevis frogs were anaesthetized in a bath solution containing 0.2% MS222 (ethyl 3-aminobenzoate methanesulfonate, Sigma-Aldrich, Taufkirchen, Germany) for 5–10 min at 20 °C. After sufficient anaesthesia was reached, ovarian lobes were obtained by partial ovariectomy [10, 78]. *X.* oocytes were injected with 50 nl RNAse-free water containing 15 to 30 ng of HA-Strep-*hTRPV3* cRNA (WPI Nanoliter 2010, World Precision Instruments, Sarasota, FL, USA) to overexpress hTRPV3. Control *X.* oocytes were injected with 50 nl RNAse-free water only. Experiments alternated strictly between hTRPV3, the bovine analogue bTRPV3, and control oocytes. This made it possible to directly compare the two TRPV3 proteins with each other. Note that the control *X.* oocytes in microelectrode and inside-out measurements were the same as in the previous study from our group in which bTRPV3 was investigated [39].

### Generation of human skin equivalents

Interfollicular primary human fibroblasts and keratinocytes were isolated from juvenile foreskin following circumcision at age 2 to 11 years (permit EA1/081/13). After cultivation for 3–4 days, they were used to generate human skin equivalents (hsEq) as described previously [42].

### Cell culture and transfection of HEK-293 cells

HEK-293 cells (DSMZ, Braunschweig, Germany, 2016/06/08) were cultivated under standard conditions (37 °C, 5% CO_2_ in humidified air) in Dulbecco’s modified Eagle’s medium (FG 0445) supplemented with 10% foetal bovine serum and 100 units · mL^−1^ of penicillin and streptomycin (all Biochrom). For seeding, the supernatant of an 80% confluent T-25 flask was removed including non-attached cells. Living cells adhering to the flask bottom were washed with phosphate-buffered saline (PBS; 5 mL; Sigma-Aldrich, St. Louis, MO, USA). After trypsinization for 5 min (1 mL; 0.05% trypsin + 0.02% EDTA (Merck, Darmstadt, Germany)), cells were incubated with trypan blue (0.4%, Sigma-Aldrich) at a ratio of 1:1 to identify non-vital cells. Viable, non-stained cells were counted manually using a hemocytometer (Paul Marienfeld GmbH & Co. KG, Germany) and a binocular inverse microscope. Subsequently, 1.2·10^6^, 6·10^5^, or 3·10^5^ HEK-293 cells were seeded into a new T-25 flask with 5 mL medium for experiments after 1, 2, or 3 days, respectively. Alternatively, cells were seeded onto coverslips for immunofluorescence staining. Polyethylenimine (PEI, linear, MW 25,000, Polysciences, Inc., Hirschberg an der Bergstrasse, Germany) was used to transiently transfect the cells with pIRES2-*AcGFP*1-HA-Strep-*hTRPV3* (hTRPV3 or wild-type), with pIRES-AcGFP1-HA-Strep-*hTRPV3*-G573S (G573S), or with the empty pIRES2-*AcGFP*1 (control) vector using the website “http://www.cytographica.com/lab/PEItransfect.html” for calculations. The medium was refreshed 24 h prior to experiments.

### Immunoblotting

Proteins were prepared, denatured, electrophoresed, and blotted onto membranes as described in supplement part B. A primary mouse antibody directed against an epitope (AA 458–474) in the first extracellular loop of hTRPV3 was used at a dilution of 1:3000 (“Anti-TRPV3”; ID: ABIN863127, antibodies-online GmbH, Aachen, Germany). This antibody was previously validated in our group for staining of the bovine homologue of TRPV3 [39]. For detection of the Strep-tag, a primary mouse antibody (“Anti-Strep”; ID: 34,850, Qiagen, Hilden, Germany) was used at the dilution of 1:2500. After blocking, the membranes were incubated with the primary antibodies (in 2.5% milk in Tris-buffered saline with Tween-20 (0.1 vol%; TBST) supplemented with NaN_3_ (0.01%)) overnight (4 °C). Horseradish peroxidase conjugated secondary antibody (anti-mouse, 1:1000 in 2.5% milk in TBST; 45 min; room temperature, Cell Signaling Technology, Frankfurt, Germany) was used to detect the primary antibodies on the membranes. Proteins were visualized by use of the Clarity Western ECL Substrate (Bio-Rad Laboratories GmbH, Munich, Germany).

### Immunofluorescence staining

All preparation steps were performed as described in [39, 73] or in the supplement part C. Anti-TRPV3 was diluted 1:1000 in goat serum (5% in PBS; PAN-Biotech GmbH, Aidenbach, Germany) to stain *X*. oocytes and HEK-293 cells (1:250 for human skin equivalents). Treated slices were incubated overnight (4 °C). Secondary antibody controls were performed with goat serum (5% in PBS) only. On the next day, slices of *X.* oocytes and human skin equivalents were incubated with Alexa Fluor® 488 conjugated goat anti-mouse IgG (1:1000; Thermo Fisher Scientific, Waltham, MA, USA) as secondary antibody (60 min, 37 °C). HEK-293 cells were stained with Alexa Fluor® 594 conjugated goat anti-mouse IgG (1:1000; Thermo Fisher Scientific) due to GFP emitting green light with peak emission at 509 nm [16]. Images were obtained using a confocal laser-scanning microscope (LSM 510, Axiovert200M, Zeiss, Jena, Germany) at 405, 488, and 543 nm.

### Double-barrelled pH-sensitive microelectrode measurements

Experiments were performed as described previously in Liebe et al. [39] and in the supplement part D. The method was used to simultaneously determine the membrane potential (U_mem_) and the intracellular pH (pH_i_) of *X.* oocytes superfused with varying solutions. Calibration was performed before and after each measurement. All experiments were performed in parallel to those on the bovine homologue (bTRPV3, [39]) at 23 °C, alternating between overexpressing *X.* oocytes and controls, with the latter also used in [39]. The following solutions were used (in mmol⋅L^−1^): “NaCl” (85 NaCl), “KCl” (81 KCl, 5 NaCl), “NaGlu” (80 sodium D-gluconate (NaGlu), 5 NaCl, 10 (2*R*,3*R*,4*R*,5*S*)-6-(Methylamino)hexane-1,2,3,4,5-pentol chloride (NMDGCl)), “NH_4_Cl” (5 NaCl, 80 NH_4_Cl), “NH_4_Cl-EDTA” (5 NaCl, 80 NH_4_Cl, 5 NMDGCl, 5 EDTA, no CaCl_2_ and no MgCl_2_), and “NMDGCl” (80 NMDGCl, 5 NaCl). In addition, all solutions contained (in mmol⋅L^−1^) 5 4-(2-hydroxyethyl)-1-piperazineethanesulfonic acid (HEPES), 1 CaCl_2_, 1 MgCl_2_, and 1 KCl and were adjusted to an osmolality of 223 mOsm · kg^−1^ (D-mannitol) and to pH 7.4 (Tris), except for “NaCl-6.4” (85 NaCl), which was adjusted to pH 6.4 and contained 5 2-(*N*-morpholino)ethanesulfonic acid (MES) instead of HEPES.

The relative permeability ratio was calculated from the membrane potentials (see supplement part E). Slopes were calculated from the regression in a 5-s interval around the point of interest.

### Inside-out patch-clamp experiments

Inside-out experiments were performed as previously described [25, 69] in a continuously perfused bath chamber at 23 °C, alternating between overexpressing *X.* oocytes and controls. Pipettes were pulled with a DMZ Universal Puller (Zeitz Instruments, Munich, Germany). Currents were recorded by an EPC 9 patch-clamp amplifier (HEKA Electronic, Lambrecht, Germany) using Patchmaster Software (HEKA Electronic). Data were sampled at 10 kHz and filtered at 250 Hz. Currents were clamped at the potentials − 60 to + 60 mV in 10 mV steps for six seconds each.

As described in Liebe et al. [39], *X.* oocytes were placed in a cell culture dish with frog oocyte Ringer (in mmol⋅L^−1^: 96 NaCl, 5 HEPES, 2.5 2-oxopropanoic acid, 1 KCl, 1 CaCl_2_, 1 MgCl_2_) to which D-mannitol was added incrementally. After dissociation of the vitelline membrane from the oolemma, it was removed with two sharpened forceps under a dissecting microscope. The stripped *X.* oocyte was then placed in a conventional flow chamber over an inverted microscope (Axiovert.A1, Zeiss) with subsequent seal formation and patch excision.

Solutions for patch-clamping were based on Doerner et al. [21] and contained (in mmol⋅L^−1^) 20 HEPES, 5 CsCl, 1 ethylene glycol-bis(β-aminoethyl ether)-*N*,*N*,*N*′,*N*′-tetraacetic acid (EGTA), and 1 KCl. The pipette and the “NH_4_Cl” bath solution additionally contained NH_4_Cl (96 mmol⋅L^−1^). In “NaCl”, “KCl,” and “NMDGCl” bath solutions, NH_4_^+^ was replaced by the same amount (96 mmol⋅L^−1^) of Na^+^, K^+^, or NMDG^+^, respectively. “NH_4_Glu” bath solution substituted 81 mmol⋅L^−1^ NH_4_Cl with NH_4_-Glu. Solutions were adjusted to an osmolality of 223 mOsm ⋅ kg^−1^ (D-mannitol) and a pH of 7.4 (Tris and HCl).

Single-channel data were analysed using Igor Pro Software (6.37, WaveMetrics Inc., Lake Oswego, Oregon, USA) as described previously [39] and in the supplement part F.

### Whole-cell experiments

Whole-cell experiments with HEK-293 cells were performed at 23 °C as previously described [39, 69] using Patchmaster Software (HEKA Electronic) with automatic correction of capacitance and series resistance. For continuous monitoring (66 s), we used a pulse protocol (“pulse protocol I”) with a low sampling rate (100 Hz), starting from a holding potential of − 40 mV and cycling from + 100 to − 120 mV in 10 mV steps. Afterwards, a protocol with a high sampling rate (5 kHz, “pulse protocol II”) was applied to assess channel kinetics. Only measurements with a series resistance between 3 and 12 MOhm were included in the evaluation. After each overexpressing HEK-293 cell, a control was measured. For analysis, type I protocols were merged.

All whole-cell solutions were adjusted to an osmolality of 300 mOsm ⋅ kg^−1^ using D-mannitol and to a pH of 7.4 with Tris and HCl. Pipette solutions for the first series of experiments in NaCl Ringer were based on Macpherson et al. [44] and contained (in mmol⋅L^−1^) 140 CsCl, 10 HEPES, 5 EGTA, and 1 MgATP. Extracellular solutions (“NaCl”) contained 140 NaCl or NMDGCl, respectively, in addition to 10 HEPES, 5 KCl, 2 MgCl_2_, and 5 EGTA. In a second series of experiments, the conductance to NH_4_^+^ was investigated using a pipette solution containing (in mmol⋅L^−1^) 120 NaGlu, 15 NaCl, 5 KCl, 5 EGTA, and 10 HEPES. Extracellular solutions in this series contained either 130 NaCl (“NaCl”) or 135 NH_4_Cl (“NH_4_Cl”) in addition to 5 KCl, 2 CaCl_2_, 2 MgCl_2_, 10 HEPES, and 10 glucose.

Stock solutions were prepared by dissolving menthol and thymol in ethanol, while 2-APB was dissolved in methanol and stored at − 20 ºC. Immediately prior to the experiments, they were added to the extracellular solution at a ratio of 1:1000 yielding the following end concentrations (in mmol⋅L^−1^): 1 menthol (2-Isopropyl-5-methyl-cyclohexanol1, racemic), 1 D-menthol (1S,2R,5S)-( +)-menthol), 1 L-menthol (1R,2S,5R)-( −)-menthol) (all from Sigma-Aldrich), 1 thymol (Carl Roth), and 0.3 2-APB (Merck).

For statistical evaluation, clamped pipette potentials were corrected for the liquid junction potential (JPCalcWin software, School of Medical Sciences, Sydney, Australia) [3]. Current densities were calculated by dividing the absolute current values obtained from pulse protocol II by the capacitance. Positive or “outward” currents reflect cations flowing out of the cell or anions flowing into it. Reversal potentials were calculated by linear interpolation between the values above and below a current of zero in the corresponding IV-curve.

### Cell viability experiments with HEK-293 cells using ruthenium red

T-25 flasks (*N* = 18) were seeded with 3·10^5^ HEK-293 cells each. After 2 days, the cells were transfected according to procedure with the wild-type hTRPV3, G573S mutant, or empty control vector (6 flasks for each vector). Afterwards, half of the flasks of each group were supplemented with ruthenium red (RR; 20 µmol⋅L^−1^; Sigma-Aldrich) for 20 h. The cell concentration in each flask was determined 4 times independently via hemocytometer as described above.

### Statistical analysis

All data were statistically evaluated using SigmaStat 11.0. After testing for normality using the Shapiro–Wilk test, comparisons between two groups were performed using the Mann–Whitney Rank Sum Test. Comparisons between three groups (see supplement) were performed using the Kruskal–Wallis One Way Analysis of Variance on Ranks followed by pairwise comparisons (Mann–Whitney Rank Sum Test). In cases where different solutions were applied consecutively, differences were evaluated using Friedman Repeated Measures Analysis of Variance on Ranks (ANOVA on Ranks) followed by the Student–Newman–Keuls method for multiple comparisons or the Wilcoxon Signed Rank Test for pairwise comparisons. A significant difference was assumed for *p* ≤ 0.05. Obtained values were given as means ± SEM, rounding as recommended by the DIN 1333 [20].

The *n* value represents the amount of individual experiments, whereas *N* refers to the number of different frogs, T-25 flasks, or donors (in the case of the skin equivalent).

## Results

### Immuno-detection of hTRPV3

To study the role of hTRPV3 in ammonia transport, HEK-293 cells were transfected with either pIRES2-*AcGFP*1-Strep-*hTRPV3*- (hTRPV3) or the empty vector (control), while *X.* oocytes were injected with pGEM-HE-MCS-Strep-*hTRPV3*-cRNA (hTRPV3) or water (control).

**Fig. 1:**
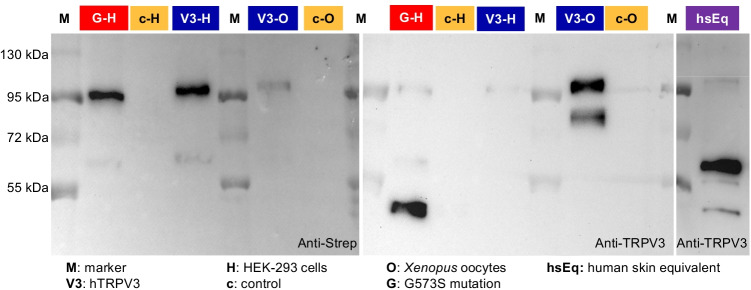
Immunoblots: detection of hTRPV3 in overexpressing HEK-293 cells, *X.* oocytes, and in human keratinocytes. Following the marker lane (M) and from left to right, lanes were loaded with protein samples extracted from G573S HEK-293 cells (1 µg; typical example of *n* = 8 replicates), control HEK-293 cells (10 ng; *n* = 19), hTRPV3 HEK-293 cells (10 ng; *n* = 14), hTRPV3 *X.* oocytes (5 µg; *n* = 7), control *X.* oocytes (5 µg; *n* = 8), G573S HEK-cells (1 µg; *n* = 11), control HEK-cells (20 ng; *n* = 15), hTRPV3 HEK-cells (20 ng; *n* = 10), hTRPV3 *X.* oocytes (1 µg; *n* = 7), control *X.* oocytes (1 µg; *n* = 7), and keratinocytes obtained from human skin equivalents (1 µg; *n/N* = 12/2). Staining with Anti-Strep, a band at the height of full-length TRPV3 (~ 95 kDa) can be seen in all overexpressing cells. At ~ 60 kDa, an additional weaker band was found in overexpressing HEK 293 cells only. Staining with Anti-TRPV3, all overexpressing cells showed the full-length band at ~ 95 kDa. *X.* oocytes expressed an additional band at ~ 80 kDa. Various breakdown products were stained at different heights in cells expressing the mutant G573S (see “Discussion” section). Control cells showed no staining. The keratinocyte protein extracted from human skin equivalents showed a weak band at ~ 95 kDa needing longer exposure times than normal in addition to various bands ≤ 60 kDa

In immunoblots (Fig. 1), staining against the Strep-tag showed a band at the predicted molecular weight of hTRPV3 (~ 95 kDa) both in HEK-293 cells overexpressing hTRPV3 (*n* = 14) or its mutant G573S (*n* = 8) and in hTRPV3 overexpressing *X.* oocytes (*n* = 7), all in line with a successful expression of the Strep-tagged protein. No staining occurred in controls for either HEK-293 cells (*n* = 19) or *X.* oocytes (*n* = 8). In HEK-293 cells only, a faint additional band appeared at ~ 60 kDa, most likely reflecting a degradation product, since it was not observed in the controls. Using the commercial Anti-TRPV3 antibody, all protein samples from overexpressing cells showed staining at the expected height of ~ 95 kDa (hTRPV3 HEK 293, *n* = 10; G573S, *n* = 11; hTRPV3 *X.* oocytes, *n* = 7). Degradation products were stained in hTRPV3 *X.* oocytes at ~ 80 kDa, with a band of similar height appearing in hTRPV3 HEK-293 cells at higher exposition times (data not shown). In G573S HEK-cells, a strong band appeared at ~ 50 kDa and a weaker one at ~ 60 kDa. Controls again showed no staining (c-HEK 293, *n* = 15; c-*X.* oocytes, *n* = 7). Note that the Strep-tag was attached to the N-terminal end of the protein, while the anti-TRPV3 antibody stained an epitope near S1-S2 in the middle of the protein (AA 458–474). This suggests that the fragments did not contain the N-terminus.

In protein from a model of human keratinocytes (human skin equivalent, 12 replicates from 2 donors), the ~ 95 kDa band was weak, requiring high exposure times. The intense band at ~ 60 kDa may correspond to a previously described splice variant first reported in human keratinocytes [74] but also found in bovine rumen [39], porcine intestine, and porcine and murine skin [46] or to a degradation product.

**Fig. 2:**
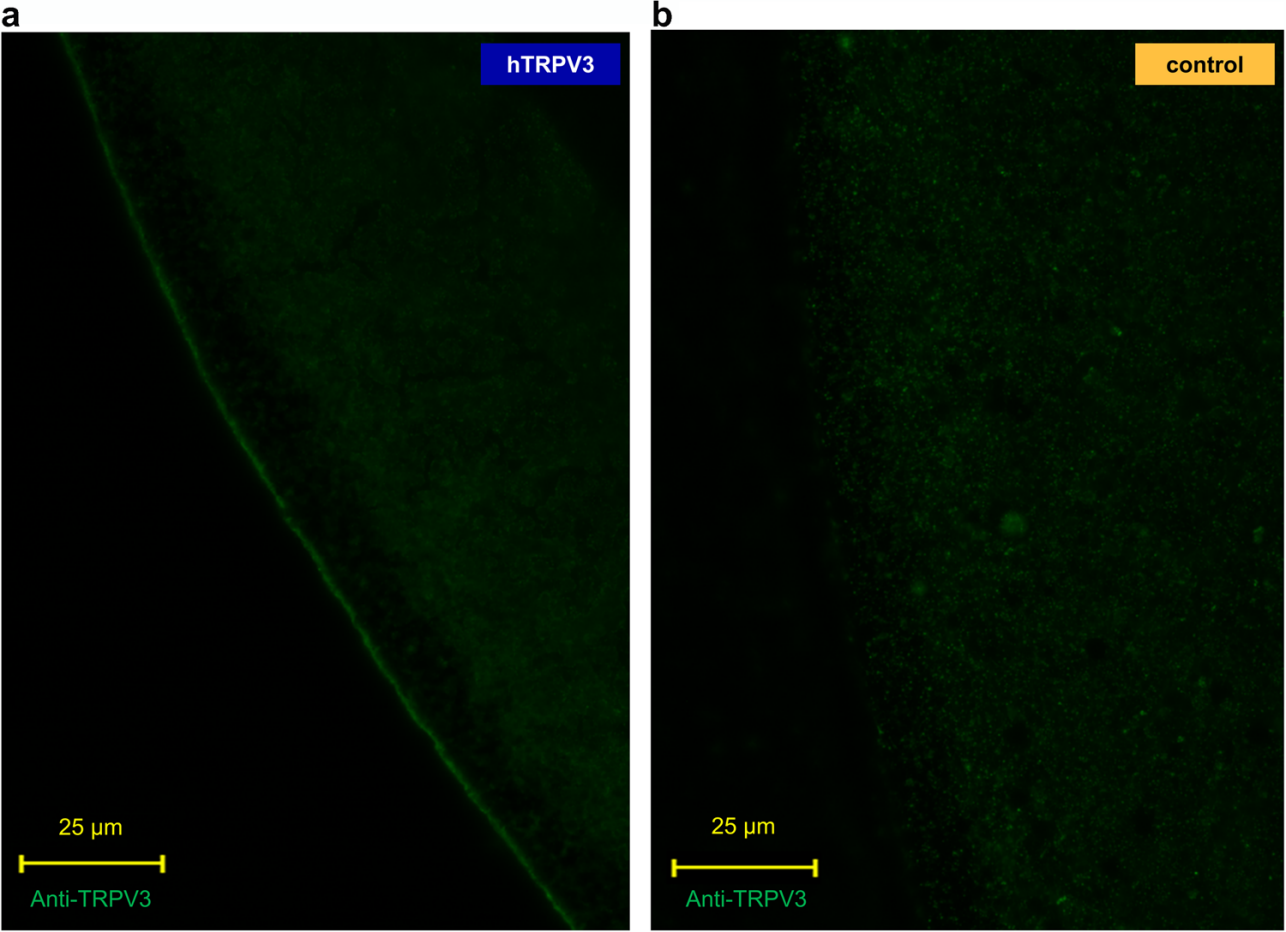
Confocal laser microscopy: localization of hTRPV3 in *X.* oocytes overexpressing hTRPV3. **a** Anti-TRPV3 distinctly stained the cell membrane of hTRPV3 *X*. oocytes (*n* = 24), confirming successful expression and trafficking. **b** Controls (*n* = 20) injected with RNA-free water showed no signal after staining with the anti-TRPV3 antibody [39]

**Fig. 3:**
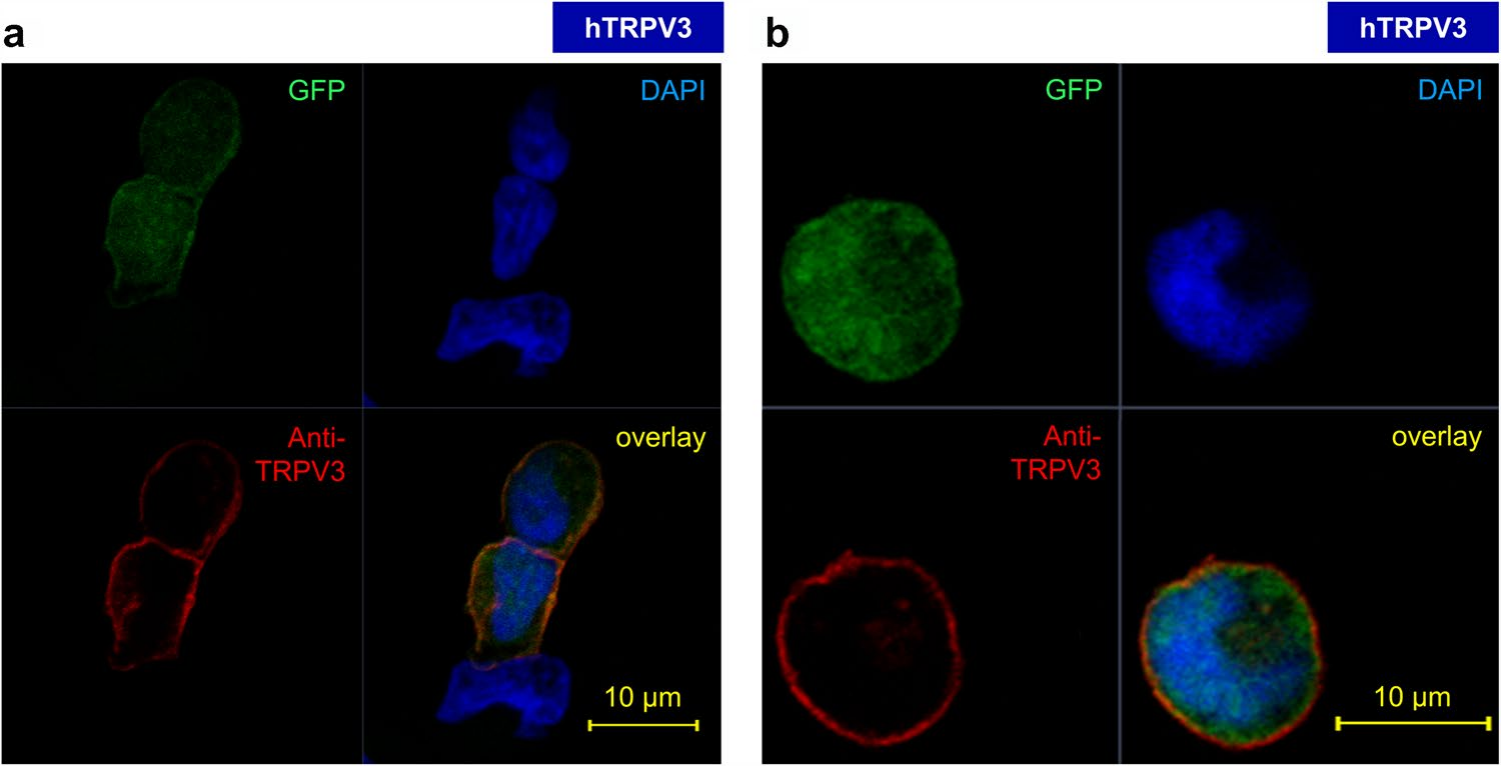
Confocal laser microscopy: localization of hTRPV3 in HEK-293 cells overexpressing hTRPV3. **a** Staining of three adjacent HEK-293 cells, two of which have been transfected successfully. The green fluorescent signal marks cells overexpressing GFP, which was expressed downstream of hTRPV3. Since the GFP protein was not fused to the channel protein, cytoplasmic staining is observed. Conversely, hTRPV3 was primarily localized to the cell membrane (Anti-TRPV3, red). Cell nuclei were stained with DAPI (blue). Cells expressing GFP also stained for hTRPV3 and unsuccessful transfection led to no staining. **b** Close-up of one isolated HEK-293 cell overexpressing hTRPV3. (number of replicates: hTRPV3 HEK-293 cells, *n* = 4 cultures; control HEK-293 cells, *n* = 12 cultures)

In immunofluorescence staining, *X.* oocytes overexpressing hTRPV3 showed membrane staining (*n* = 24), which was absent in controls (*n* = 20) (Fig. 2, green). Successfully transfected HEK-293 cells showed green fluorescence in the cytosol (*n* = 4 cultures, Fig. 3). Anti-TRPV3 (in red) primarily stained the cell membrane of overexpressing HEK-293 cells indicating unimpaired expression and trafficking of the hTRPV3 channel protein. Control cells (*n* = 12, data not shown) and non-fluorescent HEK-293 cells (Fig. 3a, bottom) failed to show staining for hTRPV3.

**Fig. 4:**
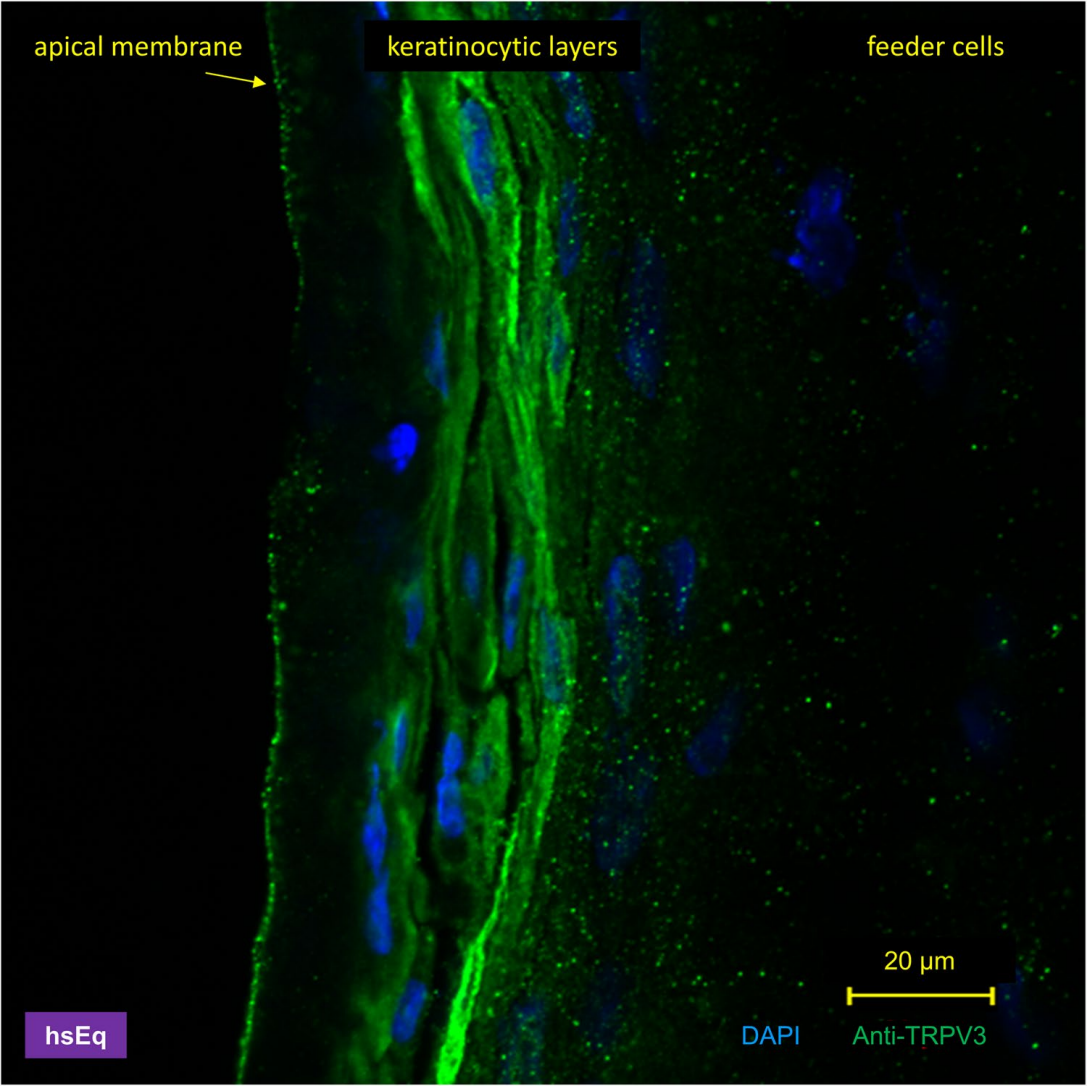
Confocal laser microscopy: localization of hTRPV3 in a human skin equivalent. Interfollicular primary human fibroblasts and keratinocytes were isolated from juvenile foreskin and grown in culture to obtain human skin equivalents, as described in detail elsewhere [42]. Staining with Anti-TRPV3 (*n* = 10) is shown in green. Cell nuclei are shown in blue (DAPI), allowing identification of the basal feeder cells (fibroblasts) to the right, which showed no staining for TRPV3. Conversely, keratinocytes showed strong staining of the cytosol, except for the top layer on the left of the image, where staining for hTRPV3 was almost exclusively observed in the apical membrane. Note that since the skin equivalent was covered with cell culture medium throughout, it did not form the equivalent of a stratum corneum

In immunofluorescence staining of the human skin equivalent (*n* = 10, Fig. 4), no staining of the fibroblasts growing on the support near the basolateral side could be observed. Strong staining of the cytosol was observed in the middle of the keratinocytic layers. Intriguingly, in the topmost layer of cells, staining was clearly visible in the apical membrane, while the cytosol was almost devoid of staining. This suggests a process of differentiation with increasing trafficking of the TRPV3 protein into the apical cellular membrane, as observed in native preparations of human skin [56]. Note that the topmost cell layer reflects an equivalent of the stratum granulosum since the human skin equivalent was immersed in medium throughout, preventing cornification.

### Experiments with pH-sensitive double-barrelled microelectrodes

Classical theories hold that NH_3_ passes into cells via lipid diffusion [54]. Since ammonia is a strong base, influx primarily in the form of NH_3_ into the cytosol should increase intracellular pH (pH_i_), with a subsequent removal of ammonia leading to a rapid steep drop in pH_i_ [49]. Conversely, influx primarily in the form of NH_4_^+^ with dissociation and subsequent removal of NH_3_ via sequestration in subcompartments, metabolism, or efflux should have an acidifying effect on pH_i_ [49]. As the system approaches the electrochemical equilibrium [50, 65], acid loading via influx of NH_4_^+^ will gradually decrease until pH regulatory mechanisms of the cell can compensate for the residual influx of protons via NH_4_^+^. Accordingly, an enhanced permeability to NH_4_^+^ should increase the speed of acidification, while the pH_i_ minimum should decrease. Simultaneously, it should be possible to observe a depolarization of the membrane potential (U_mem_), the magnitude of which will depend not on the absolute influx of NH_4_^+^ but on its magnitude relative to other conductances.

In accordance with the latter scenario, preliminary screening experiments on four hTRPV3 expressing *X.* oocytes showed that application of NH_4_Cl led to an acidification and depolarization of each oocyte studied. After washout, a very slow pH increase could be observed, clearly distinct from the rapid drop in pH associated with an efflux of NH_3_ (see supplement part H, Fig. S1). Possible reasons for the remarkably slow recovery will be discussed further down.

**Fig. 5:**
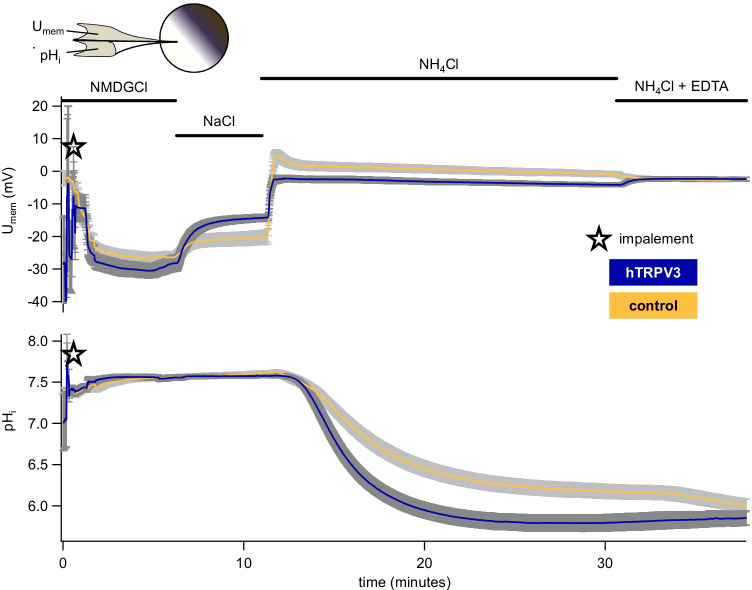
Double-barrelled pH-sensitive microelectrodes: response of control *X.* oocytes and *X.* oocytes expressing hTRPV3 to NH_4_Cl solution (means ± SEM). The blue traces show the means ± SEM of hTRPV3 *X.* oocytes (*n/N* = 12/3), while the orange traces represent the means of the controls (*n/N* = 16/3) [39], with SEM values shown in shades of grey. Impalement is marked by the star and the sharp drop in U_mem_. *X.* oocytes were incubated in NMDGCl containing solution in the days before the experiment and in its initial phase. Subsequently, the bath solution was switched to various solutions as indicated by the bars. Acidification occurred after application of NH_4_Cl with a stronger effect in hTRPV3 *X.* oocytes, reflecting greater influx of NH_4_^+^

Transport primarily in the form of NH_4_^+^ is supported by subsequent more rigorously performed investigations (Fig. 5 and Table 1). The experiments were conducted in parallel with another project [39], alternating between hTRPV3 (*n/N* = 13/3), the bovine homologue bTRPV3 (*n/N* = 14/3), and control *X.* oocytes (*n/N* = 16/3) (see supplement part H, Fig. S2 and Table S2). To prevent damage to *X.* oocytes overexpressing hTRPV3 due to influx of Na^+^, all groups of oocytes were incubated in NMDGCl solution prior to the experiment and in its initial phase. A switch to NaCl resulted in a depolarization that was significantly higher in the hTRPV3 group, reflecting greater permeability to Na^+^ with a higher p(Na^+^)/p(NMDG^+^). Conversely, the depolarizing effects of NH_4_^+^ were larger in control oocytes. This suggested that in addition to non-selective cation channels as previously assumed [11], endogenous K^+^ channels may have contributed to the NH_4_^+^ response of the control oocytes in our study [18, 33, 82]. Alternately, the permeability of the endogenous non-selective cation channels to Na^+^ may have been lower than that to K^+^. Note that U_mem_ only measured the permeability ratio relative to other ions (as indicated by the Goldman-Hodgkin-Katz equation) and not the absolute influx rate, which may be why significant differences did not emerge.

**Table 1:** Double-barrelled pH-sensitive microelectrodes: effects of NH_4_^+^ on the pH_i_ and the membrane potential of *X.* oocytes expressing hTRPV3. The table shows means ± SEM of measurements using hTRPV3 (hV3, *n/N* = 13/3) and control *X.* oocytes (ctrl, *n/N* = 16/3) consecutively exposed to various solutions. Values were measured 5 min after exposure unless indicated otherwise. The last column shows the statistical comparison of the two groups using the Mann–Whitney Rank Sum Test. Within columns, different superscripts indicate significant differences with *p* ≤ 0.05 (ANOVA on Ranks)

Bath solution	hTRPV3	Control	*p* (hV3 vs. ctrl)
Membrane potential (mV)			
NMDGCl	− 30.6 ± 2.2^a^	− 27.2 ± 1.8^a^	0.4
NaCl	− 14.4 ± 1.1^b^	− 20.3 ± 2.2^b^	0.03
NH_4_Cl (3.5 min)	− 2.5 ± 1.0^c^	1.5 ± 1.4^c^	0.03
NH_4_Cl (20 min)	− 4.1 ± 0.6^d^	− 0.9 ± 0.8^d^	0.003
NH_4_Cl-EDTA	− 2.3 ± 0.6^c^	− 2.7 ± 0.5^e^	0.7
Intracellular pH (pH_i_)			
NMDGCl	7.56 ± 0.04^a^	7.56 ± 0.04^a^	0.9
NaCl	7.58 ± 0.03^a^	7.61 ± 0.03^b^	0.5
NH_4_Cl (3.5 min)	6.76 ± 0.09^b^	7.14 ± 0.06^c^	0.004
NH_4_Cl (20 min)	5.81 ± 0.11^c^	6.18 ± 0.09^d^	0.007
NH_4_Cl-EDTA	5.85 ± 0.09^c^	6.05 ± 0.08^e^	0.07
Change of pH_i_ (slope in ΔpH/min)			
NMDGCl	0.03 ± 0.02^a^	0.01 ± 0.01^a^	0.5
NaCl	0.00 ± 0.00^a^	0.00 ± 0.01^a^	0.6
NH_4_Cl (3.5 min)	− 0.39 ± 0.04^b^	− 0.22 ± 0.04^b^	0.006
NH_4_Cl (20 min)	0.02 ± 0.01^a^	0.00 ± 0.01^a^	0.05
NH_4_Cl-EDTA	− 0.004 ± 0.02^a^	− 0.04 ± 0.02^c^	0.01
Relative permeability ratio p(X) / p(NMDG^+^)			
Ion X			
Na^+^	1.98 ± 0.18^a^	1.37 ± 0.12^a^	0.005
NH_4_^+^ (20 min)	2.93 ± 0.24^b^	2.93 ± 0.23^b^	1.0
NH_4_^+^ (in EDTA)	3.15 ± 0.25^c^	2.70 ± 0.18^c^	0.3

Influx of NH_4_^+^ is confirmed by analysing the pH_i_ data. Application of NH_4_^+^ induced a strong acidification with a final pH_i_ in NH_4_Cl solution that was significantly lower in hTRPV3 oocytes than in controls, confirming a higher permeability to NH_4_^+^ in the hTRPV3 group (Fig. 5 and Table 1). Prior to NH_4_Cl application, an alkaline drift was observed that led to a significant difference in the absolute pH_i_ values between NaCl and NMDGCl. It is tempting to speculate on an involvement of NHE, but the lack of an impact of the solution change on the slope of the pH_i_ curve indicates that any involvement of endogenous NHE was weak (Fig. 5 and Table 1).

In both groups, the speed of acidification after application of NH_4_^+^ was initially high with influx driven both by the negative membrane potential and a high concentration gradient for NH_4_^+^, but levelled off as the oocytes approached an equilibrium distribution (Table 1 and Fig. 5). Twenty minutes after application of NH_4_^+^, the pH_i_ of hTRPV3 oocytes started to recover slightly, suggesting a situation near equilibrium. Accordingly, opening of hTRPV3 channels after removal of Ca^2+^ showed no significant effect on pH_i_ (*p* = 0.2). Conversely, the rate of acidification increased after application of EDTA in control oocytes, suggesting that equilibrium had not been reached. Most likely, removal of Ca^2+^ opened endogenous NH_4_^+^ permeable non-selective cation channels [11, 82].

The effects of a removal of Ca^2+^ on the membrane potential were inverse in hTPRV3 and control oocytes. All hTRPV3 oocytes depolarized significantly with a significant rise in p(NH_4_^+^)/p(NMDG^+^), suggesting a greater influx of NH_4_^+^ through hTRPV3 channels that were opened by the removal of Ca^2+^. Conversely, all control oocytes hyperpolarized. *X.* oocytes express rather unique endogenous Cl^−^ channels that open when Ca^2+^ is removed [61, 82]. It appears likely that in control oocytes, the corresponding hyperpolarization overrode the opening of endogenous non-selective cation channels, while in oocytes overexpressing hTRPV3, the effect on hTRPV3 channels predominated. Note that the corresponding relative permeability ratios (which were calculated from the membrane potentials) contain contributions of this Ca^2+^ inactivated Cl^−^ conductance and must therefore be considered with caution.

In conjunction, these results suggest that both groups of *X.* oocytes expressed conductances to Na^+^ and NH_4_^+^, but that permeability to Na^+^ was greater and influx of NH_4_^+^ more rapid in hTRPV3 oocytes than in controls. In both groups, any permeability to NH_3_ was much smaller than that to NH_4_^+^. The comparison with the bovine homologue [39] is shown in the Supplement (part H and Fig. S2) and will be discussed below.

### Inside-out patch-clamp experiments

In total, patches from 30 hTRPV3 and 21 control *X.* oocytes from three frogs were investigated, alternating between hTRPV3, bTRPV3, and control *X.* oocytes [39]. In all groups, there was a tendency for single-channel events to occur in one solution and vanish in another without apparent reason. Channel activity mostly rose with the duration of the experiment.

**Fig. 6:**
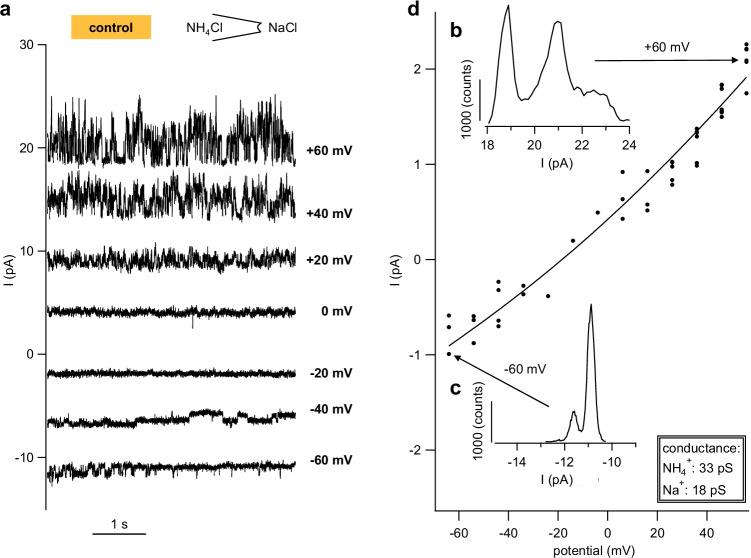
Single-channel recordings of a patch from a control *X.* oocyte showing channels permeable to NH_4_^+^ and Na^+^. This measurement was performed in the inside-out configuration with NaCl in the bath (facing the cytosolic side) and NH_4_Cl in the pipette (external side), with pipette potentials as indicated. **a** Original recording showing channel events that were visibly smaller at negative potentials (influx of Na^+^ from the bath) than at positive potentials (efflux of NH_4_^+^ from the pipette). **b** Amplitude histogram of the trace at + 60 mV. The distance between the two large peaks was used to determine the unitary conductance. The noisy residue above 22 pA most likely reflects very brief openings of additional channels. **c** Amplitude histogram of the trace at −60 mV, the peak distance is smaller than in (**b**). **d** All unitary currents (black circle) from this patch were plotted against the potential. The data were fitted with a GHK fit (black line) yielding a higher conductance for NH_4_^+^ than Na^+^ (also see the histogram in Fig. 9)

The pipette solution contained NH_4_Cl, and the bath was changed from NaCl to NH_4_Cl and NH_4_Glu. In control *X.* oocytes, 14 out of 21 patches showed single-channel activity in at least one solution (supplement, Fig. S4). Unitary events were not altered by the replacement of Cl- by the larger anion, Glu^−^, arguing against an anion conductance. In an asymmetrical configuration with NaCl in the bath, channel openings were visible at negative potentials, reflecting influx of Na^+^. At positive potentials, larger channel openings could be observed, reflecting efflux of NH_4_^+^ (Fig. 6a). The results of the GHK-analysis (Fig. 6b) varied from patch to patch. The mean conductance of this diverse group of channels was previously reported in Liebe et al. [39] (43 ± 9 pS for NH_4_^+^ (*n* = 13) and 33 ± 10 pS for Na^+^ (*n* = 11)).

**Fig. 7:**
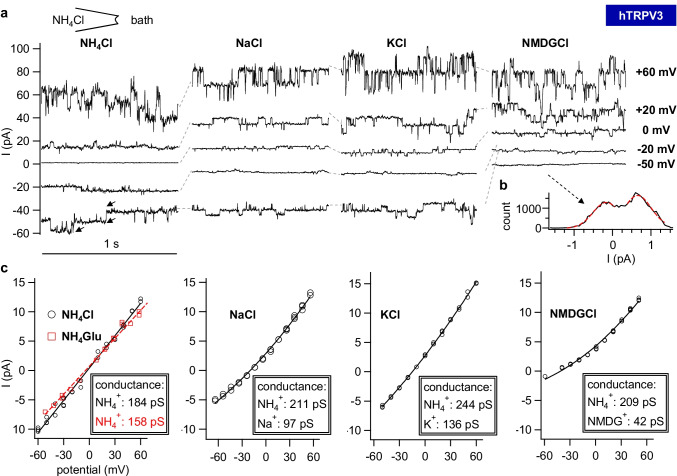
Single-channel recordings of a patch from a hTRPV3 *X.* oocyte in symmetrical NH_4_^+^ solution. This measurement was performed in the inside-out configuration with NH_4_Cl in the pipette and consecutively applied bath solutions as indicated. **a** Traces at pipette potentials of + 60 mV, + 20 mV, 0 mV, −20 mV, and −50 mV. In symmetrical NH_4_Cl solution at −50 mV, the arrows indicate three different current levels corresponding to the opening of two channels. Channel openings of similar size were visible at positive and negative potentials. In NMDGCl, reduction in the amplitude of channel openings was clearly visible at −50 mV with channel activity reduced to a flicker. The absolute current level also dropped sharply suggesting that much of the baseline current in the previous traces was channel-mediated. Conversely, at +60 mV, unitary currents remained at the same level, reflecting efflux of NH_4_^+^ from the pipette into the bath. **b** Amplitude histogram of the trace at −50 mV in NMDGCl solution. The red broken lines represent two Gauss fits. The distance between their peaks was used to estimate the unitary current. **c** Unitary currents (white circle) from amplitude histograms as exemplarily in (**b**) were plotted over the potential. A linear fit was used for data from NH_4_Cl solution (black) and NH_4_-Glu solution (red, no traces shown above). All other data were fitted with the GHK equation for two cations to yield the conductances as indicated (also see the histogram in Fig. 9)

**Fig. 8:**
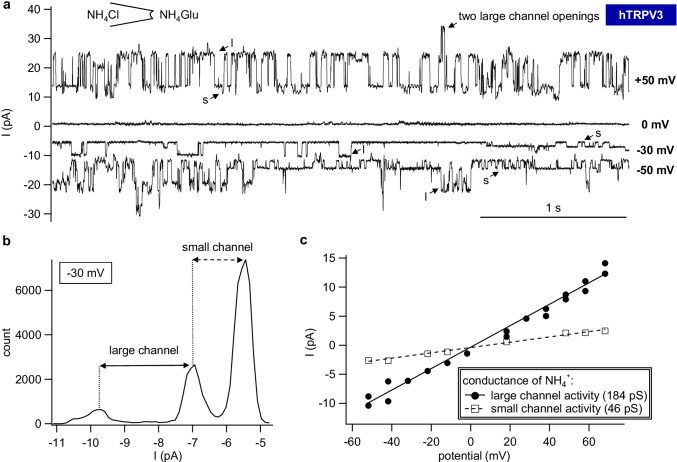
Single-channel recordings of a patch from an hTRPV3 *X.* oocyte showing both endogenous channels and hTRPV3. This measurement was performed with NH_4_Cl in the pipette and NH_4_Glu in the bath. **a** At +50 mV, 0 mV, −30 mV, and −50 mV pipette potential, both large (l) and small (s) channel openings occurred in the same trace as indicated by the arrows. At 0 mV, there is no electrochemical gradient for NH_4_^+^, and no channel activity could be observed. At +50 mV, two large channels opened at the same time leading to a higher current response. **b** In the amplitude histogram corresponding to the trace shown in Fig. 8a at −30 mV, three peaks were clearly visible with unitary currents indicated by the double arrows. The small channel was usually open, while the large channel had a much lower open probability but higher unitary current. **c** All unitary currents were plotted against the clamped potentials. The data of large and small channels were fitted separately to yield the conductances for NH_4_^+^

Of 30 hTRPV3 patches, six showed no channel activity (supplement Fig. S4). In total, hTRPV3 patches showed a channel conductance in NH_4_Cl solution that was three times higher than in controls (132 ± 68 pS *n/N* = 19/3, *p* = 0.001). The sharp decline of current at negative potentials in NMDGCl solution suggests that much of the baseline current in the other traces reflects continuously open cation channels (Fig. 7). Five patches showed small channel events with a conductance (G) for NH_4_^+^ of 62 ± 49 pS (*p* = 0.6 vs. control). Ten other patches showed both large and small channel events, sometimes appearing simultaneously in the same trace (Fig. 8). In nine patches, activity of channels was initially lacking or small with G(NH_4_^+^) < 100 pS, although later, larger channels with G(NH_4_^+^) > 100 pS became visible.

**Fig. 9:**
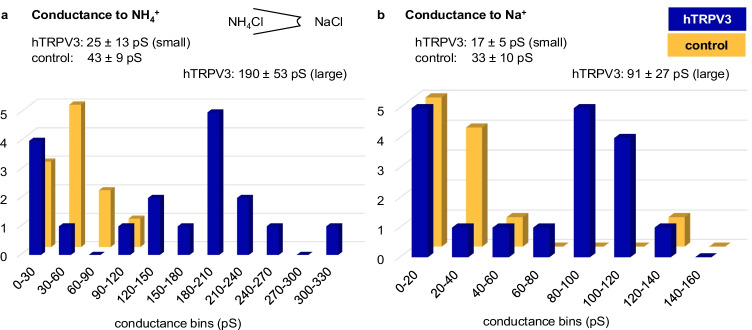
Inside-out patch-clamp measurements: histograms of all NH_4_^+^ and Na^+^ conductances from *X*. oocytes. The conductances visualized in the histograms were obtained in asymmetrical configuration with NH_4_Cl in the pipette and NaCl in the bath. The vertical axis yields the number of patches in which the conductance fell into a particular conductance bin as shown on the horizontal axis. **a** All NH_4_^+^ conductances from control patches (orange) were below 100 pS. Patches of hTRPV3 *X*. oocytes showed both small NH_4_^+^ conductances such as those observed in controls and larger NH_4_^+^ conductances ranging up to 303 pS, possibly reflecting formation of heteromers of hTRPV3 with endogenous channels. **b** Corresponding histogram of all Na^+^ conductances. Although channels were smaller, a similar pattern emerged as in (**a**), with a cluster of channels below 60 pS in both groups and a second larger cluster emerging in hTRPV3 *X.* oocytes. Interestingly, one solitary control patch expressed a very large conductance to Na^+^ reflecting what is clearly a very diverse population of channels expressed by the native *X*. oocyte [82]. Note that these measurements were performed in oocyte Ringer (96 mmol·L^−1^), yielding lower conductances than would have been expected in mammalian Ringer (145 mmol·L^−1^)

Since channel events yielding a conductance > 100 pS were almost exclusively observed in hTRPV3 *X.* oocytes, they were considered to reflect hTRPV3 channel activity (Fig. 9), with the scatter possibly reflecting formation of heteromers with endogenous channels. The conductance of these larger channels was G(Na^+^) = 91 ± 27 pS and G(NH_4_^+^) = 190 ± 53 pS. A further histogram in the supplement includes bTRPV3 conductances (Fig. S3). These values were obtained in oocyte Ringer, which contained lower concentrations of Na^+^ and NH_4_^+^ than the standard Ringer solutions used when working with HEK-293 cells. Assuming that the independence principle applies, the corresponding values for 145 mmol·L^−1^ can be obtained using Eq. 4 in supplement part F, yielding G(Na^+^) = 137 ± 42 pS and G(NH_4_^+^) = 287 ± 80 pS, respectively.

### Whole-cell patch-clamp experiments

#### NaCl

**Fig. 10:**
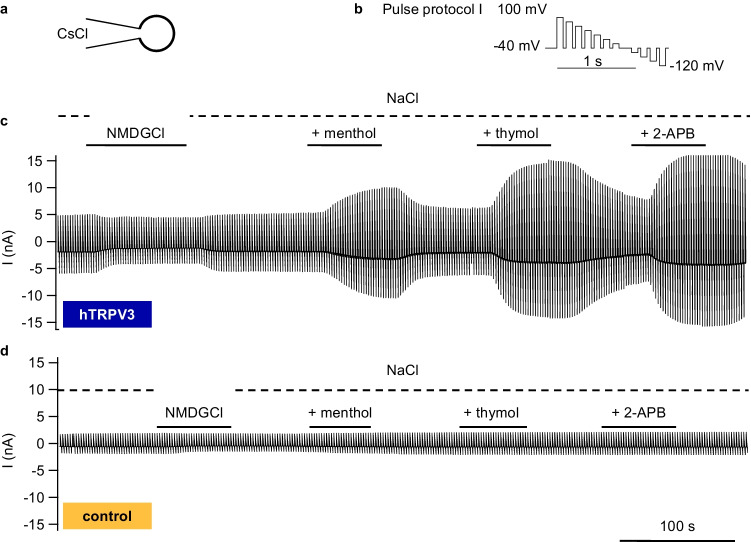
Whole-cell recordings: response of hTRPV3 HEK-293 cells to TRP channel agonists in NaCl solution. To confirm functional expression of hTRPV3, measurements were performed in a standard configuration using divalent-free solutions with CsCl in the pipette and NaCl in the bath (**a**). Cells were stimulated with the continuous pulse protocol I shown in (**b**). Pulse protocols were merged to visualize whole-cell currents measured in one hTRPV3 HEK-293 cell (**c**) and a control cell (**d**). Both cells showed a reduction in inward current at negative pipette potentials when extracellular Na^+^ was exchanged for the poorly permeable NMDG^+^, indicating the presence of cation channels. After return to NaCl, the three TRPV3 agonists menthol (1 mmol·L^−1^), thymol (1 mmol·L^−1^), and 2-APB (1 mmol·L^−1^) were consecutively applied with significant effects in overexpressing cells, while no observable impact was detectable in controls. Washout significantly reduced the currents (hTRPV3, *n* = 19; control, *n* = 13, see also Table 2)

Functional expression of hTRPV3 in HEK-293 cells was investigated using a CsCl pipette solution and a Ca^2+^- and Mg^2+^-free NaCl bath solution [44]. After stabilization of current, agonists of hTRPV3 [44, 79] were applied, namely, menthol (racemic form; 1 mmol·L^−1^), thymol (1 mmol·L^−1^), and 2-APB (0.3 mmol·L^−1^), with subsequent NaCl-washout (Fig. 10 and Table 2).

**Table 2:** Whole-cell measurements: effects of menthol, thymol, and 2-APB on Na^+^ currents and the reversal potential of HEK-293 cells expressing hTRPV3. The table shows means ± SEM of whole-cell measurements of hTRPV3 (hV3, *n* = 19) and control (ctrl, *n* = 13) HEK-293 cells consecutively exposed to various solutions. Both the pipette solution (CsCl) and the bath solution (NaCl) were divalent-free resulting in large current densities. Numbers in parentheses reflect repeated application of the same bath solution. The last column gives the *p* values between the two groups (Mann–Whitney Rank Sum Test). Different superscripts indicate significant differences (*p* ≤ 0.05) between different solutions within one group (ANOVA on Ranks)

Bath (pipette: CsCl)	hTRPV3	Control	*p* (hV3 vs. ctrl)
Outward current (pA · pF^−1^) at + 100 mV			
NaCl	135 ± 24^a^	64 ± 22^a^	0.04
NMDGCl	145 ± 29^a^	57 ± 21^b^	0.01
NaCl (2)	141 ± 26^a^	60 ± 21^a^	0.03
Menthol in NaCl	327 ± 99^b^	64 ± 22^a^	0.001
NaCl (3)	172 ± 34^c^	67 ± 22^a^	0.03
Thymol in NaCl	575 ± 139^d^	67 ± 23^a^	≤ 0.001
NaCl (4)	338 ± 106^e^	69 ± 24^a^	0.004
2-APB in NaCl	1058 ± 140^f^	70 ± 24^a^	≤ 0.001
NaCl (5)	615 ± 115^d^	70 ± 26^a^	≤ 0.001
Inward current (pA · pF^−1^) at − 120 mV			
NaCl	− 129 ± 28^a^	− 67 ± 26^a^	0.16
NMDGCl	− 85 ± 20^b^	− 47 ± 19^b^	0.22
NaCl (2)	− 111 ± 26^c^	− 61 ± 22^a^	0.18
Menthol in NaCl	− 255 ± 88^d^	− 66 ± 25^a^	0.05
NaCl (3)	− 129 ± 29^a^	− 71 ± 26^a^	0.14
Thymol in NaCl	− 481 ± 123^e^	− 72 ± 27^a^	≤ 0.001
NaCl (4)	− 306 ± 112^d^	− 72 ± 27^a^	0.05
2-APB in NaCl	− 993 ± 122^f^	− 75 ± 28^a^	≤ 0.001
NaCl (5)	− 634 ± 130^e^	− 102 ± 39^a^	≤ 0.001
Reversal potential (mV)			
NaCl	− 12.0 ± 4.6^a^	− 8.2 ± 2.8^a^	0.9
NMDGCl	− 27 ± 4.2^b^	− 21.8 ± 4.4^b^	0.4
NaCl (2)	− 8.9 ± 1.3^c^	− 10.4 ± 4^a^	0.8
Menthol in NaCl	− 10.9 ± 2.4^d^	− 7.9 ± 2.5^a^	0.4
NaCl (3)	− 10.3 ± 3.6^ae^	− 7.3 ± 2.7^a^	0.7
Thymol in NaCl	− 10.1 ± 1.1^c^	− 7.7 ± 2.8^a^	0.05
NaCl (4)	− 13.3 ± 3.8^c^	− 8.3 ± 3.2^a^	0.3
2-APB in NaCl	− 18.5 ± 4.2^f^	− 7.6 ± 2.8^a^	0.002
NaCl (5)	− 11.7 ± 2.8^c^	− 7.8 ± 3.0^a^	0.1

In the initial NaCl solution, hTRPV3 cells (*n* = 19) showed a higher outward current density at + 100 mV than the controls (*n* = 13), reflecting a higher efflux of Cs^+^ through hTRPV3. When Na^+^ was replaced by NMDG^+^, current at − 120 mV in controls changed by − 18 ± 7 pA·pF^−1^, slightly lower than in hTRPV3 cells (− 44 ± 10 pA·pF^−1^, *p* = 0.06). the relative permeability ratios (p(Na^+^)/p(NMDG^+^)) were not different at 1.8 ± 0.2 (controls) and 1.9 ± 0.3 (hTRPV3) (*p* = 0.8). As reported by Macpherson et al. [44], highly significant differences between the two groups emerged after exposure to the agonists (Table 2).

#### NH_4_Cl

In subsequent experiments, the permeability to NH_4_^+^ was investigated with a NaGlu pipette solution. Cells were initially superfused with a NaCl solution, with equal concentrations of Na^+^ inside and outside. The external solution contained physiological amounts of divalent cations, leading to significantly lower currents than in the Ca^2+^- and Mg^2+^-free solutions used above (Tables 2 and 3).

**Table 3:** Whole-cell measurements: effects of D-menthol on NH_4_^+^ currents and the reversal potential of HEK-293 cells expressing hTRPV3. The table shows means ± SEM of measurements of hTRPV3 (hV3) and control (ctrl) HEK-293 cells consecutively exposed to various solutions that contained physiological amounts of Ca^2+^ and Mg^2+^, resulting in smaller current densities than in Table 2. Numbers in parentheses reflect repeated application of the same bath solution. The last column gives the *p* values between the two groups within the same solution (Mann–Whitney Rank Sum Test). Different superscripts indicate significant differences (*p* ≤ 0.05) between values measured in different solutions within the same cell type (ANOVA on Ranks)

Bath (pipette: NaGlu)	hTRPV3	*n*	Control	*n*	*p* (hV3 vs. ctrl)
Outward current (pA · pF^−1^) at + 100 mV					
NaCl	9 ± 3^a^	18	6 ± 2^a^	12	0.9
NH_4_Cl	16 ± 4^b^	18	17 ± 3^b^	12	0.3
NH_4_Cl + D-menthol	116 ± 42^c^	18	20 ± 3^b^	12	0.01
NH_4_Cl (2)	98 ± 44^d^	16	19 ± 2^c^	10	0.7
NaCl (2)	86 ± 42^b^	16	10 ± 2^d^	10	0.3
Inward current (pA · pF^−1^) at -120 mV					
NaCl	− 11 ± 4^a^	18	− 5 ± 2^a^	12	0.4
NH_4_Cl	− 21 ± 6^b^	18	− 9 ± 2^b^	12	0.6
NH_4_Cl + D-menthol	− 102 ± 62^c^	18	− 10 ± 3^b^	12	0.05
NH_4_Cl (2)	− 85 ± 42^c^	16	− 13 ± 4^c^	10	0.2
NaCl (2)	− 67 ± 38^b^	16	− 9 ± 3^d^	10	0.5
Reversal potential (mV)					
NaCl	− 1 ± 7^a^	18	10 ± 7^a^	12	0.2
NH_4_Cl	19 ± 5^b^	18	26 ± 4^b^	12	0.2
NH_4_Cl + D-menthol	15 ± 2^b^	18	28 ± 4^b^	12	0.01
NH_4_Cl (2)	14 ± 3^b^	16	27 ± 5^b^	10	0.11
NaCl (2)	− 1 ± 5^a^	16	19 ± 7^a^	10	0.03

**Fig. 11:**
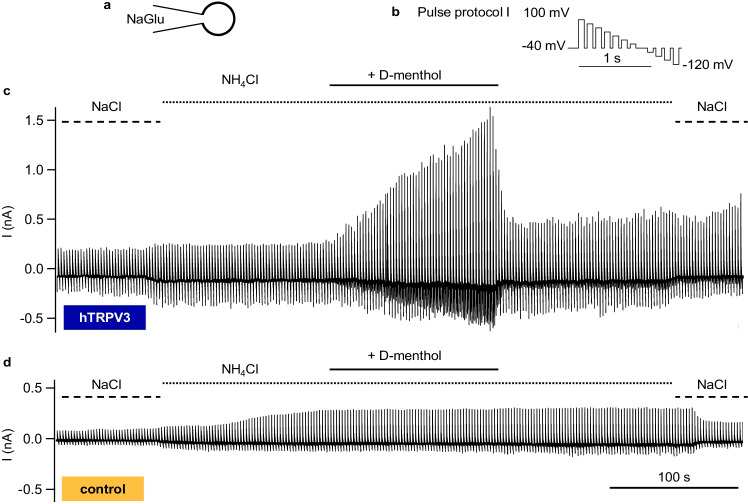
Whole-cell recordings: effects of D-menthol on NH_4_^+^ mediated currents in HEK-293 cells. Measurements were performed with a NaGlu pipette solution (**a**) using pulse protocol I (**b**) with physiological concentrations of divalent cations in the bath solution. Merged pulse protocols show the current responses for one hTRPV3 HEK-293 cell (**c**) and for one control cell (**d**). Note the increase in inward current at negative potential in both groups after the change from NaCl to NH_4_Cl. In hTRPV3 expressing cells, but not in controls, application of D-menthol (1 mmol·L^−1^) strongly increased both inward current (carried by NH_4_^+^) and outward current (carried by Na^+^). Washout significantly reduced the currents (hTRPV3, *n* = 18; control, *n* = 12; see also Table 3)

**Fig. 12:**
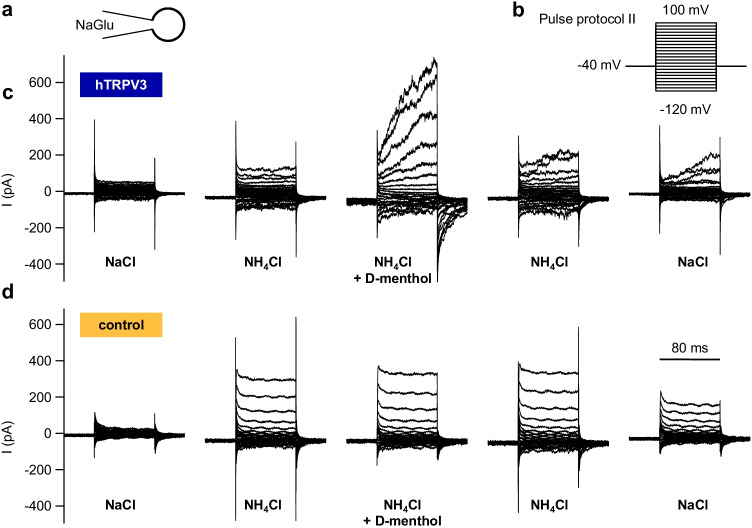
Whole-cell recordings: kinetics of NH_4_^+^ currents in HEK-293 cells. Measurements were performed with cells filled with NaGlu pipette solution (**a**) using pulse protocol II (**b**). Cells were superfused with NaCl, NH_4_Cl, supplemented D-menthol in NH_4_Cl, and washout, consecutively. **c** Original recording of a HEK-293 cell overexpressing hTRPV3. Note the typical current kinetics after application of D-menthol (1 mmol·L^−1^) with strong current activation at depolarizing potentials and subsequent tail currents after return to −40 mV. These tail currents reflect NH_4_^+^ that is entering the cell via previously opened hTRPV3 channels. **d** Original recording of a control cell, showing a strong induction of outward current in response to application of NH_4_Cl, but no further stimulation by D-menthol (hTRPV3, *n* = 18, control, *n* = 12; see also Table 3)

In the initial NaCl solution, any difference between hTRPV3 and control cells did not test for significance (Figs. 11 and 12 and Table 3). In both cell types, a switch to NH_4_Cl bath solution resulted in a significant rise in inward current level at − 120 mV in conjunction with a significant depolarization of the reversal potential, reflecting influx of NH_4_^+^. A concomitant rise in outward current at + 100 mV was observed (see discussion). From the reversal potentials, relative permeability ratios were calculated, yielding ratios for p(NH_4_^+^)/p(Na^+^) of 2.3 ± 0.5 (hTRPV3) and 2.0 ± 0.3 (control) (*p* = 0.9).

In hTRPV3 cells only, application of D-menthol led to a significant rise in inward and outward currents, reflecting an activation of hTPRV3 channels with influx of NH_4_^+^ (at − 120 mV) and efflux of Na^+^ (at + 100 mV). Additionally, relative permeability p(NH_4_^+^)/p(Na^+^) rose significantly to 3.1 ± 0.7 (*p* < 0.05). In controls, p(NH_4_^+^)/p(Na^+^) remained at 2.5 ± 0.6 (*p* > 0.05) with no significant difference between groups. The hTRPV3 currents had interesting kinetics with a time-dependent activation after depolarization and tail currents after a return to the holding potential of − 40 mV (Fig. 12), reflecting influx of NH_4_^+^ that decreased with time. Most likely, both the activation at high positive potentials followed by tail currents after repolarization were caused by voltage-dependent interaction with external Ca^2+^. Note that internal Ca^2+^ was buffered by EGTA, preventing an internal block by Ca^2+^.

Interestingly, in two control cells, a reversible reduction in inward current level could be observed. L-menthol inhibits cardiac L-type channels [4], which have a certain pharmacological similarity to endogenous Ca^2+^ channels expressed by HEK-293 cells [7].

**Fig. 13:**
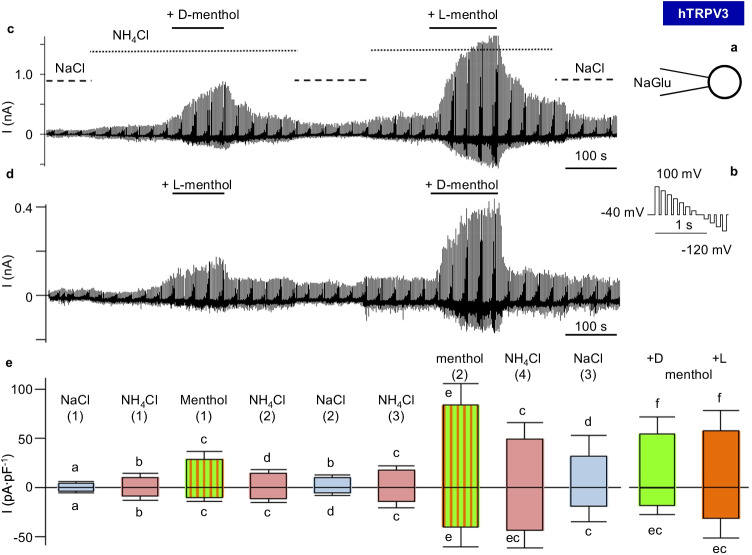
Whole-cell recordings: comparison of the effects of D- and L-menthol on NH_4_^+^ currents in HEK-293 cells. In order to compare the effects of two menthol enantiomers on NH_4_^+^ currents through hTRPV3, measurements were performed with cells filled with NaGlu pipette solution (**a**) using pulse protocol I (**b**) and bath solutions as indicated. The order in which D-menthol (1 mmol·L^−1^) and L-menthol (1 mmol·L^−1^) were applied was switched after every successful experiment. **c** Original recording of one of 5 hTRPV3 HEK-293 cells first exposed to D-menthol and then to L-menthol. **d** Original recording of one of 5 hTRPV3 HEK-293 cells first exposed to L-menthol and then to D-menthol. **e** Statistical evaluation (boxplot). The numbers in parentheses reflect repeated application of the same bath solution so that “menthol (1)” designates the response to the first application of menthol as either D- or L-menthol, while “menthol (2)” designates the response to the second application of either enantiomer. The two boxes in green (“ + D”) and red (“ + L”) show separate evaluation of data for each of the two enantiomers. Different letters indicate significant differences (*p* < 0.05) within the same group (*n* = 10, ANOVA on Ranks)

While effects of L-menthol on TRPV3 are classical [44], we are not aware of studies using D-menthol. In order to compare the effects of D- and L-menthol, experiments were performed in which both agonists were consecutively applied. The sequence of the application of the two forms of menthol was altered between experiments so that five cells were first treated with D-menthol and then with L-menthol, while in further 5 cells, the order was inverse (Fig. 13c,d). While the effects of the second application of menthol were always significantly larger, no difference between the two enantiomers emerged (Fig. 13e).

### Investigation of the Olmsted mutant G573S of hTRPV3

**Fig. 14:**
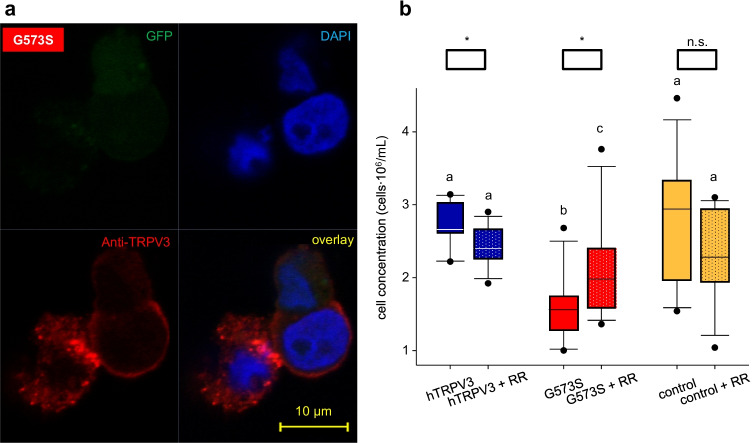
Expression of the Olmsted mutant G573S in HEK-293 cells. **a** HEK-293 cells expressing the hTRPV3 mutant G573S were immunostained 22 h after transfection (for details, see supplement part G (*n* = 26)). As in Fig. 3, DAPI (blue) was used to stain the cell nuclei. All three cells showed staining with the anti-TRPV3 antibody (red), with one cell showing clear trafficking of the protein to the cell membrane. Conversely, the HEK-293 cell that can be seen on the left showed strong cytosolic staining. Using transmitted-light microscopy, signs of a general structural degradation with partial loss of the cell membrane were observed in this cell. The GFP (green), which should be co-expressed as in Fig. 3, was too low for detection in all three cells. **b** Boxplots show the cell viability of hTRPV3, G573S, and control HEK-293 cells. Half of the passages were incubated with the TRP antagonist RR (*n/N* = 12/3). A significantly lower number of cells transfected with G573S (red) was found to be intact in comparison to cells transfected with hTRPV3 (blue) and controls (orange). Supplementing RR, cell viability was lower in the hTRPV3 and control groups and significantly higher in G573S. Different letters indicate significant differences determined using ANOVA on Ranks with Student–Newman–Keuls method. For the effect of RR, differences were determined using Mann–Whitney Rank Sum Test (*:*p* < 0.05; n.s.: not significant)

Since conflicting reports are found in the literature, a final goal of the current study was to investigate whether the G573S mutation of hTRPV3 (G573S) is trafficked to the cell membrane [22, 40]. Immunoblots suggested successful but weak expression of G573S in HEK-293 cells (Fig. 1). Individual G573S HEK-293 showed Anti-TRPV3 immunofluorescence staining in the cytosol. Even fewer cells showed staining within the cell membrane (Fig. 14a). However, it should be stressed that in the 26 cultures investigated, only very few cells could be shown to have any staining for G573S, and of those that did, most deviated morphologically from non-expressing controls, and all failed to express significant amounts of GFP. Furthermore, large numbers of detached and deformed cells were found floating in the supernatant. In order to enhance the viability of HEK-293 cells expressing the mutant, we attempted various approaches, which included reducing the concentration of free Ca^2+^ in the medium, changing the concentration of foetal bovine serum or altering incubation times after transfection that are summarized in supplement part G. All of these attempts were unsuccessful.

Transfection with the *G573S* vector resulted in a significantly reduced HEK-293 cell count in comparison to wild-type hTRPV3 (*p* ≤ 0.001; Fig. 14b). In a next step, the TRP antagonist ruthenium red (RR) was added to the medium, which should block channels from the external side. In cells expressing G573S, RR induced a significant (*p* = 0.04) increase in the cell count. Conversely, the wild-type hTRPV3 cell count was significantly reduced by RR (*p* ≤ 0.05). Note that the effect of RR on G573S cells was significantly (*p* ≤ 0.001) stronger in comparison to hTRPV3 cells. Cell counts of controls responded to RR in a similar way as wild-type hTRPV3, although the difference induced by ruthenium red was not significant (*p* = 0.2). Despite the RR treatment, the number of successfully transfected G573S cells remained very low, and staining for GFP was insufficient. For this reason, attempts to patch-clamp HEK-293 cells with the G573S mutation of hTRPV3 failed.

## Discussion

Using the whole-cell and inside-out configuration of the patch-clamp technique and double-barrelled pH-sensitive microelectrodes in two different expression systems, the present study clearly shows that the human homologue of TRPV3 channel (hTRPV3) conducts NH_4_^+^. It may be argued that this is hardly surprising in light of the low ability of TRPV3 to discriminate between different cations [58] and our previous findings concerning the bovine homologue [39], but a conductance of hTRPV3 to NH_4_^+^ has to be shown rigorously before any further deliberations are possible. Parts of the study were performed in a manner to allow a direct comparison between the human and the bovine TRPV3, but the data of this study do not support a significant functional difference between the two homologues.

In a further step, we demonstrated that both the native hTRPV3 of human keratinocytes and the OS mutant G573S are trafficked to the cellular membrane, in line with established models [17, 52, 53]. However, there are also signs of an expression in intracellular organelles. We conclude that while hTRPV3 is certainly important for Ca^2+^ signalling as classically proposed [17, 52], it may also mediate relevant exchanges of monovalent cations such as NH_4_^+^ both between organelles and the cytoplasm and between the cytoplasm and the extracellular space, with consequences for nitrogen exchanges and protein metabolism.

It may be argued that ammonia can simply diffuse through the lipid membrane, so why is a transport protein needed? However, ammonia (NH_3_) is a very polar molecule which strongly binds H^+^ so that at physiological pH, over 98% is found in the form of ammonium (NH_4_^+^). Both the strong dipole moment and the low concentrations of NH_3_ argue against sizable transport rates via lipid diffusion. Instead, the strong alkalinization observed in many types of cells exposed to NH_4_Cl reflects expression of Rhesus-like glycoproteins (AMT/Rh) [30] and aquaporins [9], which catalyse the deprotonation of NH_4_^+^, so that NH_3_ is formed, which then passes through these protein pores into the cell [51]. In other preparations, uptake occurs primarily as ammonium (NH_4_^+^). In the thick ascending loop of Henle, uptake of ammonium occurs via a K^+^ channel (ROMK) [18] and the NKCC cotransporter, which is not surprising since the hydration radii and, thus, the hydration energies of NH_4_^+^ and K^+^ are similar [36]. In native *X.* oocytes [11, 39] and the ruminal epithelium, non-selective cation channels have emerged as the primary uptake route for NH_4_^+^ [1, 8, 59, 66]. Since uptake is driven by the negative membrane potential, this mechanism should be particularly useful if nitrogen needs to be scavenged for synthesis of non-essential amino acids. This is certainly the case in ruminants on traditional diets, but it is also possible to speculate that *X.* oocytes might profit from being able to take up ammonia from degradation processes within a pond.

### Detection of the protein

In the current study, hTRPV3 channels were expressed in HEK-293 and *X.* oocytes. Immunoblots (Fig. 1) with staining against the Strep-tag showed bands at the predicted height (~ 95 kDa) in the hTRPV3 and G573S overexpressing cells, but not in controls. A weak band (~ 60 kDa) could be seen in the hTRPV3 and G573S HEK-293 cells, most likely reflecting a degradation product since it was not seen in the controls. This band was not seen either in *X.* oocytes or in our previous study of HEK-293 cells expressing the bovine TRPV3 for reasons that are unclear [39].

A similar pattern emerged after staining overexpressing HEK-293 cells and *X.* oocytes with Anti-TRPV3. In addition to the ~ 95 kDa band, a second strong band was observed in overexpressing *X*. oocytes at ~ 80 kDa. Again, no staining could be observed in the controls, arguing against non-specific effects. Although a weak band could be seen at ~ 95 kDa, the most prominent band of the G573S HEK-293 cells had a molecular weight of ~ 50 kDa followed by several lower weight bands. Possibly, these bands reflect breakdown products within apoptotic cells. Cleavage of the N-terminally placed Strep-tag would explain why these bands could not be seen in the immunoblots stained with Anti-Strep. At the exposure times used for the expressing systems, only the ~ 60 kDa band was visible in proteins from human keratinocytes, although at high exposure times, the full-length protein (~ 95 kDa) could be detected in addition to some smaller bands (Fig. 1). In our previous study of the rumen [39] and in an unrelated study of cultured keratinocytes [74], the ~ 60 kDa band was also stronger than the ~ 95 kDa band, although the difference in staining intensity was not as pronounced. Furthermore, we observed that the ~ 60 kDa band was always more pronounced than the ~ 95 kDa band in various parts of the porcine intestine, the porcine skin and in murine skin [46]. While this band may reflect a breakdown product, sequences for a ~ 60 kDa splice variant of TRPV3 are available for the bovine species (AAI46079.1) and for mice (XP_006533411.1) and suggest that the protein is truncated after amino acid 527 (mouse) or 526 (bovine) (for alignment, see the supplement of [46]). This is in the middle of the third transmembrane domain (S3) so that the pore region (S5 and S6, > aa580) is missing [81]. It thus appears unlikely that the short variant functions as an ion channel, but it may have regulatory properties that clearly need to be explored.

In immunofluorescence staining, hTRPV3 was clearly targeted to the cell membrane in hTRPV3 overexpressing cells (Figs. 2 and 3), which, in the case of HEK-293 cells, coincided with the expression of cytosolic GFP. Control HEK-293 cells and control *X.* oocytes showed no staining so that the antibody appears specific for hTRPV3. The functional expression of hTRPV3 in HEK-293 cells was cross-checked using whole-cell experiments demonstrating currents sensitive to menthol, thymol, and 2-APB in overexpressing cells, but not in controls (Fig. 10 and Table 2) [44]. The permeability of hTRPV3 to NH_4_^+^ was unambiguously shown using both expression systems and three different experimental approaches.

### Experiments on X. oocytes

A first series of experiments was performed using *X.* oocytes as a well-established expression system [13, 26]. Experiments were performed both with pH-sensitive microelectrodes and with the single-channel configuration of the patch-clamp technique. Experiments on the bovine homologue of TRPV3 (bTRPV3) were conducted in parallel and published previously [39]. Comparative results are shown in the supplement (parts H and I).

In both overexpressing oocytes and controls, experiments with pH-sensitive microelectrodes showed a depolarization and a clear acidification of the cytosol after incubation with NH_4_^+^ (Fig. 5 and Table 1) with a slow recovery of pH_i_ after washout (Fig. S1). Despite their frequent use as a system for studying ammonia transport, it thus appears that native *X.* oocytes amply express endogenous pathways for the uptake of NH_4_^+^. This finding confirms previous reports [11, 35, 49] and may have contributed to the controversy surrounding the preferred substrates of members of the AMT/Rh transporter family [51].

The response of native *X.* oocytes to NH_4_^+^ containing solutions was first studied in depth by Burkhardt and Frömter [11]. These authors demonstrated that NH_4_^+^ produced a depolarization and acidification that could not be blocked by either K^+^ channel blockers such as Cs^+^ or TEA (triethylamine) or by blockers of NKCC such as bumetanide [11]. Furthermore, the removal of NH_4_Cl did not lead to the anticipated rapid acidification of the cytosol due to efflux of NH_3_, but to a pH recovery towards a more alkaline pH. The authors concluded that the *X.* oocyte membrane was almost completely impermeable to NH_3_ and that, instead, NH_4_^+^ was being taken up via non-selective cation channels. These observations were basically confirmed in a later publication by Keicher and Meech [35], although in that study, evidence emerged that transporter expression varied with the stage of oocyte maturation.

In what is arguably the most in depth study of the issue by Musa-Aziz et al. from the Boron laboratory [49], *X.* oocytes were impaled by one electrode for the membrane potential (U_mem_) and by another for pH_i_, while a third blunt pH electrode was pushed against the oocyte from the outside so that surface pH (pH_s_) could additionally be monitored. Although a depolarization was again observed, it was much slower than in the current study or previous ones [11, 35, 39] for reasons that are not clear. Again as described previously, an intracellular acidification was observed. Paradoxically, pH_S_ also dropped. The authors concluded in their excellent discussion that NH_3_ was clearly entering the oocyte. However, they had no definite explanation for the NH_4_^+^ induced changes in pH_i_ and U_mem_. They also pointed out that pH_s_ data predicted a flux ratio of *J*_NH3_/*J*_NH4_^+^  > 2/100, while pH_i_ data supported *J*_NH3_/*J*_NH4_^+^  < 0.5/100. These contradicting fluxes are clearly impossible unless the *X.* oocyte is surrounded by two separate membranes. Indeed, *X*. oocytes are surrounded not only by a lipid membrane (or oolemma), but also by a stabilizing network of protein fibres (the vitelline membrane) that is permeable to water and solutes. A removal of the vitelline membrane was not reported [49] and does not appear likely, since this would have made the oocyte preparation too unstable for manipulation with three electrodes (own observations). We suggest that diffusion of the small NH_3_ molecule through the vitelline membrane should be more rapid than that of the heavily hydrated NH_4_^+^ ion, explaining the changes in pH_s_ [49]. In a subsequent step, NH_4_^+^ could then leave the perivitelline space and pass into the cytosol via a channel of the oolemma, explaining the changes in pH_i_ and U_mem_ measured by the cytosolic electrodes.

Another important insight to emerge from the study of Musa-Aziz et al. [49] is that incubation of *X.* oocytes in as little as 0.5 mmol·L^−1^ NH_4_Cl solution for 30 min leads to an accumulation of cytosolic [NH_3_/NH_4_^+^] to a level of over 3.3 mmol·L^−1^. The U_mem_ was at ~  − 40 mV, and the pH_i_ was at 7.17 so that the cytosolic concentration of NH_4_^+^ clearly exceeded the equilibrium concentration of ~ 2.4 mmol·L^−1^ for NH_4_^+^ alone (with the contribution of NH_3_ to total ammonia negligible). Conversely, no evidence for glutamine synthesis could be found by Musa-Aziz et al. after a 10-min incubation [49] so that this aspect does not contribute to the observations within the timeframe of these experiments.

In conjunction, a relatively consistent model emerges. While the vitelline membrane appears to be more permeable to NH_3_ than to NH_4_^+^, the inverse is true for the lipid bilayer surrounding the *X.* oocyte, which is, in fact, almost impermeable to NH_3_. The presence of NH_4_^+^ permeable channels in the oolemma is clearly supported the changes in pH_i_ and U_mem_ and by our single-channel data. Within the cytosol, a fraction of the NH_4_^+^ dissociates into a proton and NH_3_, with the latter sequestered in intracellular vesicles [49]. Acidification will slow down as the system approaches the equilibrium distribution for NH_4_^+^. After washout, repolarization is almost immediate, but the alkalinization will be much slower than the initial acidification for a number of reasons. Firstly, the release of NH_3_ trapped in the intracellular stores may require time [49]. Secondly, while uptake of NH_4_^+^ is supported by the electrical gradient, efflux of NH_4_^+^ from the repolarized oocyte has to occur against the electrical gradient. Thirdly, many non-selective cation channels (such as TRPV3) are outwardly rectifying so that the permeability of the channel is lower at negative than at positive potentials. For this reason, the rate of efflux of NH_4_^+^ from the repolarized oocyte in NaCl solution will be slower than the rate of influx measured after depolarization by application of NH_4_Cl. Functionally, the native *X.* oocyte is thus able to scavenge ammonia–nitrogen, which might be useful for protein synthesis.

In the current study, initial values of pH_i_ (7.56) and U_mem_ (~ − 30 mV) of hTRPV3 *X.* oocytes were similar to those of the control oocytes (Table 1 and Fig. 5). Previous studies of native *X.* oocytes have reported a pH_i_ of ~ 7.5 [12] and U_mem_ within the range of − 40 to − 60 mV [11, 35, 49]. The higher U_mem_ in the current study possibly reflects the fact that after injection, the *X.* oocytes were incubated in NMDGCl solution. This approach was adopted to avoid excessive influx of Na^+^ in the hTRPV3 oocytes, but it may have compromised uptake of K^+^ by the Na^+^/K^+^-ATPase in both groups. The initial rate of acidification after application of NH_4_^+^ was significantly higher in hTRPV3 oocytes than in controls (Table 1). A slight recovery could be observed after 20 min, most likely reflecting a situation at the equilibrium point at which net influx of NH_4_^+^ had ceased so that pH regulatory mechanisms became effective [65]. Since control oocytes did not acidify as quickly, the pH_i_ was significantly higher after 20 min than in oocytes overexpressing hTRPV3. It appears that at this point, residual NH_4_^+^ influx and the pH regulatory mechanisms were roughly in balance so that no acidification and no recovery of pH_i_ was observed.

Apart from these differences, a very striking difference versus controls was the inverse response to replacement of Ca^2+^ by EDTA (Fig. 5 and Table 1). This manoeuvre opens many non-selective cation channels, including TRPV3. For hTRPV3 *X.* oocytes, removal of Ca^2+^ induced no change in pH_i_, which is to be expected if NH_4_^+^ is at equilibrium [65]. Conversely, control oocytes, which had obviously not yet reached the Nernst equilibrium for NH_4_^+^, began to acidify again. In line with this hypothesis, all hTRPV3 *X.* oocytes depolarized significantly, reflecting the opening of TRPV3 channels. Unexpectedly, all control *X.* oocytes hyperpolarized. This most likely reflects the expression of rather unique endogenous Cl^−^ channels that open when Ca^2+^ is removed [61, 82]. We suggest that in the hTRPV3 expressing oocytes, this effect was obscured by the larger effect due to the opening of hTRPV3.

For inside-out experiments, the vitelline membrane was removed to expose the lipid membrane of the *X.* oocyte, which subsequently had to be handled very carefully. The experiments clearly demonstrate the presence of non-selective cation channels with a permeability to NH_4_^+^ in both native *X.* oocytes and in the oocytes overexpressing hTRPV3. However, channels with G(NH_4_^+^) > 100 pS were only found in the hTRPV3 *X.* oocytes (Figs. 7, 8, and 9). Conductance levels were very variable, most likely explained by formation of heteromers with endogenous channels expressed by the *X*. oocyte. Intra-family heteromerization of TRPV3 with other TRP channels has long been known [71].

Single-channel conductances were comparable, while acidification of hTRPV3 was stronger than in bovine TRPV3. This probably reflects a higher level of expression in the hTRPV3 *X.* oocytes rather than a more fundamental functional difference (supplement, Fig. S4).

In whole-cell experiments, both hTRPV3 HEK-293 cells and controls expressed channels permeable to NH_4_^+^ with a significant increase in influx at − 120 mV pipette potential and a higher reversal potential (Table 3). Outward current at + 100 mV also increased. This may reflect efflux of Na^+^, influx of Cl^−^, or a combination of both, induced by changes in pH_i_ or swelling. Furthermore, an increase in current is frequently seen when the concentration of a permeant ion rises [33]. Significant differences in NH_4_^+^ influx emerged after hTRPV3 cells and controls were stimulated by application of D-menthol (( +)-menthol) (Figs. 11 and 12 and Table 3). Cells overexpressing hTRPV3 not only showed different current amplitudes but also different current kinetics (Fig. 12 and Table 3). The most likely explanation is that depolarization repels positively charged Ca^2+^ and Mg^2+^ ions from the mouth of the hTRPV3 channel pore (voltage-dependent block by divalent cations [39, 55, 58]) leading to the time-dependent increase in current observed at positive potentials. At negative potentials, the inverse happens and influx of NH_4_^+^ decreases as divalent cations are drawn into the channel. In line with this explanation, strong tail currents followed the depolarizing pulses, reflecting an influx of NH_4_^+^ that decreased as more and more divalent cations returned to the channel mouth. Furthermore, we showed that D-menthol and L-menthol had similar effects on hTRPV3 so that the activation mechanism appears to be independent of the chirality (Fig. 13). As observed previously, pre-activation of the channel by either form increased the subsequent response to the agonist [44], despite washout between the applications.

One goal of the study was to investigate the G573S mutant, which causes Olmsted syndrome (OS) with hyperkeratinization in humans. This dominant mutation is localized in the linker region between the S4 and S5 segment of the TRPV3 subunit, interfering with normal channel gating so that in the mutant, the channel is locked in an open conformation [22, 40, 81]. In one previous study, expression of OS-mutants in HaCaT cells caused impaired vesicular trafficking that resulted in reduced surface localization of these hTRPV3 mutants and other membrane proteins [85], suggesting that Olmsted syndrome might be primarily a lysosomal disorder. Conversely, other studies using HEK-293 cells have reported successful membrane expression of G573S [40] and a number of other OS associated mutants [87] in the cell membrane. We confirm that at least when expressed in HEK-293, the G573S mutation can be trafficked to the membrane in individual cells (Fig. 14a). However, it was also clearly apparent that the number of cells that showed staining was extremely low and of these few cells, many showed marked staining within the cytosol and severely impaired structural morphology. Interestingly, none of the cells showed visible staining for GFP, which is in marked contrast to the observations using the wild-type hTRPV3 or bTRPV3 construct. The most likely explanation for these observations is that the expression of the mutant G573S induced cell death immediately after insertion of the channel protein in the membrane and before sufficient expression of GFP could occur.

The problem of significantly increased death rate in cells expressing gain of function hTRPV3 mutants was described earlier by Lin et al. [40] and most likely reflects apoptosis or necrosis due to Ca^2+^ influx [64]. This may explain why despite extensive attempts (supplement part G), we were unable to obtain a sufficient number of cells that successfully co-expressed GFP to identify G573S cells for patch-clamping. Possibly, this problem might have been prevented using a vector with fusion of G573S to GFP, as in the study by Lin et al. [40]. On the other hand, we purposely avoided this approach since the fusion of the channel to a large marker protein may be one reason why the mutant channel was not correctly trafficked to the membrane in the study of Yadav et al. [85]. Furthermore, we were concerned that fusion of GFP to the mutant channel might alter its properties. In this context, it is possible to speculate that a decrease in conductance due to the fusion protein enhanced cell viability in the study of Lin et al. [40]. As reported by this group, we confirm that expression of G573S severely impairs cell survival and that cells can be partially rescued by ruthenium red (RR) (Fig. 14b) [40]. RR is a large cation that is thought to block the extracellular mouth of the mutant channel, thus preventing influx of cations. These experiments support the hypothesis that expression in the extracellular membrane is crucial to the ability of G573S to cause cell death under in vitro conditions.

Staining of a skin equivalent consisting of human fibroblasts and keratinocytes clearly showed that expression of hTRPV3 primarily occurs in keratinocytes with expression in the apical membrane of the top layer of cells (Fig. 4). Skin equivalents from cultured human keratinocytes are increasingly used as human-based test systems for basic and preclinical research [42]. Such skin equivalents do not express cells of neuronal origin, and it is possible to obtain very thin preparations, ensuring good optical properties for confocal laser microscopy. Since the construct was kept covered with cell culture medium throughout, the stratum corneum was not formed, which might be useful for future transport studies. Cells in the middle of the keratinocyte layer showed strong staining of the cytosol, most likely reflecting expression of hTRPV3 by the endoplasmic reticulum, as previously reported not just for hTRPV3 [85], but also for many other TRP channels [32]. Cells in the top layer showed a strikingly hTRPV3-free cytosol with staining for hTRPV3 almost exclusively visible in the apical membrane, suggesting a function in the apical uptake of ions. On the whole, the expression pattern of hTRPV3 in the skin equivalent remarkably resembled that found in native human skin [56] or the rumen [39]. In the colonic epithelium, which consists of only one layer of cells, staining for hTRPV3 is also localized primarily within the apical membrane [46, 75], with very little cytosolic staining. Notably, both the colonic and the ruminal epithelium functionally express a divalent-sensitive conductance to NH_4_^+^ that can be stimulated by agonists of TRPV3 [46, 66].

**Fig. 15:**
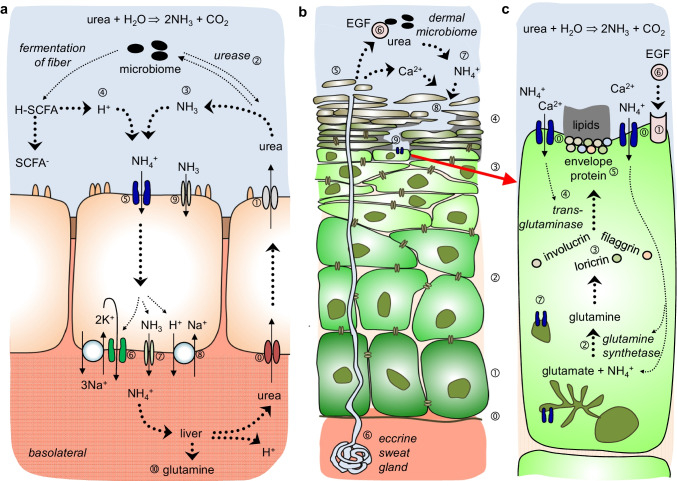
Model: TRPV3—an epithelial ammonium transporter? **a** Simplified model of nitrogen recycling across the colonic epithelium. While mammalian enzymes cannot break down urea to salvage the nitrogen, large quantities enter the colonic lumen via transporters such as UT-B, UT-A6, or aquaglyceroporins (⓪ and ①). Within the colonic lumen, the resident microbiome produces ureases that break up urea, forming ammonia (NH_3_, ③). Bacterial enzymes also degrade complex carbohydrates, forming short-chain fatty acids (H-SCFA) that release protons (④). Removal of these protons is important for colonic homeostasis. One possibility is absorption of NH_4_^+^ via electrogenic pathways that include TRPV3 (⑤). Basolateral efflux may involve K^+^ channels (⑥) or Rhesus-like glycoproteins such as RhBG (⑦) coupled to basolateral pH regulatory mechanisms such as NHE (⑧) or Na^+^-HCO_3_^-^-cotransporters. Conversely, efflux from the lumen in the form of NH_3_ (e.g. via apical RhCG (⑨)) will not remove protons from the lumen. Within the liver, ammonia can be used for the formation of urea for colonic buffering. In particular, in situations where dietary intake of protein is low, synthesis of glutamine and other non-essential amino acids may enhance survival (⑩). **b** Simplified model of human skin. The basement membrane (⓪) is followed by the cells of the stratum basale (①). Differentiation of these cells consecutively yields the stratum spinosum (②), stratum granulosum (③), and the stratum corneum (④). The cytosolic compartment of the fully differentiated corneocytes is filled with water and electrolytes, but lacks cell nuclei and organelles, while the lipid membrane is replaced by a protein envelope that binds intercellular lipids, forming a tight barrier separating the inside of the body from the outside. In shedding (⑤), the corneodesmosomes that adjoin the corneocytes are lysed so that desquamation can occur. Hyperkeratosis occurs when formation of corneocytes exceeds desquamation. Primarily in humans, thermoregulation involves formation of eccrine sweat that contains Na^+^, Cl^-^, Ca^2+^, urea, and epidermal growth factor (EGF; ⑥). Urea is degraded to NH_4_^+^ (⑦) by urealytic bacteria that colonize the skin. Small defects in the stratum corneum (⑧) provide access to the apical membrane of the stratum granulosum (⑨). **c** Detail from (**b**) showing an apical cell from the stratum granulosum. The apical membrane of the top layer of the stratum granulosum expresses TRPV3 channels (⓪), which form a signalling complex with the epidermal growth factor receptor (①). EGF, Ca^2+^, and NH_4_^+^ from eccrine sweat enter through defects in the *stratum corneum*. Entry of NH_4_^+^ facilitates cytosolic formation of glutamine from glutamate and NH_4_^+^ (②). Glutamine is required for the synthesis of involucrin, loricrin, and filaggrin (③). Entry of Ca^2+^ via TRPV3 activates the transglutaminases (④) that are required for the cross-linking of these proteins, forming the corneocyte envelope (⑤). EGF (⑥) regulates the open probability of TRPV3, fine-tuning the process. Especially in the middle of the stratum spinosum and stratum granulosum, TRPV3 is also expressed by cytosolic structures (⑦), which may reflect recruitment of channels in vesicles for trafficking to the apical membrane or other functions

So what role might a very promiscuous channel with remarkably low selectivity to Ca^2+^ play in the apical membrane of an epithelium? Certainly, uptake of Ca^2+^ is important—in the skin, to activate the transglutaminases; in the case of the rumen, to supply the animal with Ca^2+^; and in the colon or the caecum, for reasons that may include inflammatory signalling. However, it is interesting to note that all three epithelia also secrete large quantities of urea, a product that is degraded by the microbiota living on the surface, yielding ammonia [2, 5, 72, 83] (Fig. 15).

In the case of the skin, more work clearly needs to be done. However, some speculation is possible (Fig. 15 b and c). After secretion of urea with sweat and degradation by the dermal microbiome [68, 77], NH_4_^+^ is released. Small lesions in the stratum corneum may facilitate access to the apical membrane of the underlying stratum granulosum, signalling a need for local replacement of corneocytes. What follows has already been outlined: Influx of NH_4_^+^ through TRPV3 stimulates the formation of glutamine from glutamate, allowing synthesis of proteins important for cornification such as filaggrin, involucrin, and loricrin [19]. Influx of Ca^2+^ via TRPV3 activates transglutaminases, which catalyse the cross-linking of involucrin and loricrin, forming the corneocyte envelope [17, 23]. Activation of the TRPV3 signalling complex by EGF—which intriguingly is also contained in eccrine sweat [67]—completes the picture. The importance of a cytosolic production of glutamine from NH_4_^+^ is highlighted by the pathophysiology of newborns with an inherited systemic deficiency of glutamine synthetase, which leads to numerous lethal defects that include necrolytic erythema of the skin [29]. Whether or not excessive influx of NH_4_^+^ via TRPV3 plays any role in OS remains to be determined, but it is interesting to note that the full-fledged clinical picture is found only in humans and there, mostly in the palms and the soles of the feet [22]. Humans produce more eccrine sweat than other mammals, with secretion highest in the palmo-plantar skin.

In the case of the colon, the function of TRPV3 is clearer (Fig. 15a). Within the colonic lumen, microbes utilize the nitrogen contained in amino acids and urea for protein synthesis, but also set free large quantities of ammonia that are absorbed into the portal blood [41, 72, 80]. In evolutionary terms, the absorption of nitrogen in the form of ammonia from the gut must be considered advantageous, since it can be re-utilized for the synthesis of urea and non-essential amino acids such as glutamine in situations where protein intake is low [72]. In part, this may certainly occur in the form of NH_3_ (e.g. via the ammonia transporter RhCG [30, 51]). However, uptake in the form of NH_4_^+^ via divalent sensitive cation channels such as TRPV3 has also been observed [46]. Uptake in the protonated form should help with the pH homeostasis of the colonic lumen, which is challenged by acids set free in the fermentation process [6]. On the downside, well-known problems arise when ammonia cannot be detoxified by the liver in hepatic disease [2, 5, 72]. In this context and others, identifying the proteins that mediate transport of ammonia represents a first step in finding new options for intervention.

## Supplementary Information

Below is the link to the electronic supplementary material.

Supplementary file1 (PDF 1392 kb)

## Data Availability

All data generated and/or analysed during the current study are available from the corresponding author on reasonable request.

## Code availability

Available on reasonable request from F.S.
