# Supplementary material for: Beyond Ca2+ signalling: the role of TRPV3 in the transport of NH4+
Source: Pflugers Arch. 2021 Oct 19;473(12):1859–84. doi: 10.1007/s00424-021-02616-0 (PMC8599221; doi:10.1007/s00424-021-02616-0)
Supplement: Supplementary file 1 — Supplementary file1 (PDF 1392 kb) [file 424_2021_2616_MOESM1_ESM.pdf]

Online Resource

Beyond Ca^{2+} signalling: a role for TRPV3 in the transport of NH_4^+

Pflügers Archiv – European Journal of Physiology

Hendrik Liebe[1,2], Franziska Liebe[1], Gerhard Sponder[1], Sarah Hedtrich[3], Friederike Stumpff[1]
stumpff@zedat.fu-berlin.de

[1]Institute of Veterinary Physiology, Freie Universität Berlin, Berlin, Germany

[2]Department of Biology, Chemistry, and Pharmacy, Freie Universität Berlin, Germany

[3]Faculty of Pharmaceutical Sciences, University of British Columbia, Vancouver, Canada

A) Harvesting and processing of *Xenopus laevis* oocytes 2

B) Preparation for immunoblotting 2

D) Double-barrelled pH-sensitive microelectrode measurements 3

E) Relative permeability ratio 4

F) Analysis of Inside-out patch-clamp data via Goldman-Hodgkin-Katz theory 4

G) Attempts to enhance cell viability of HEK-293 cells overexpressing G573S 5

H) Supplemental microelectrode data: hTRPV3, bTRPV3, and control *X.* oocytes 6

I) Comparison of single-channel patch-clamp data: hTRPV3, bTRPV3, and control *X.* oocytes 11

A) Harvesting and processing of *Xenopus laevis* oocytes

Xenopus laevis oocytes (*X.* oocytes) were obtained and prepared as described by Vitzthum *et. al*. [11] and in [1]. After surgical removal, ovarian lobes were placed in oocyte Ringer's solution (180 mOsm·kg^{-1} adjusted with D-mannitol), shaken mechanically for 90 minutes, and transferred into calcium-free oocyte Ringer's solution for 10 minutes. Defolliculated stage V-VI *X.* oocytes were stored in oocyte culture solution at +16 °C until the following day, when they were injected (see methods). Injected *X.* oocytes were incubated for at least three days in modified low-sodium oocyte Ringer's solution (NMDGCl Ringer solution, 16 °C) before use in experiments (in mmol·L^{-1}: 80 N-methyl-D-glucamine chloride (NMDGCl), 5 NaCl, 5 4-(2-hydroxyethyl)-1-piperazineethanesulfonic acid (HEPES), 2.5 2-Oxopropanoic acid, 1 KCl, 1 $CaCl_2$, 1 $MgCl_2$, 50 units·mL^{-1} penicillin, 0.05 mg·mL^{-1} streptomycin, pH 7.4 adjusted with tris(hydroxymethyl)aminomethane (Tris), 223 mOsm·kg^{-1} adjusted with D-mannitol).

B) Preparation for immunoblotting

Both the solvents and the samples were cooled throughout the experiments to minimize protein degradation and processed essentially as in Liebe *et al* [7]. In brief, injected *X.* oocytes were incubated for four days, lysed mechanically in oocyte lysis buffer (500 µL; in mmol·L^{-1}: 5 $MgCl_2$, 5 NaH_2PO_4, 1 EDTA, 80 sucrose, pH 7.4 (Tris)), and centrifuged at 200 rpm (10 min, 4 °C). The supernatant was centrifuged using the same settings, and subsequently, at 13,000 rpm (40 min, 4 °C). The precipitate was suspended in fresh oocyte lysis buffer (40 µL). Cultivated to confluence in a T-25 flask, HEK-293 cells were washed with PBS, harvested by scraping, and centrifuged (500 g, 5 min). The cell pellet was suspended in PBS (1 mL), centrifuged (700 g, 4 min) and resuspended in RIPA buffer (100 µL). Lysis was performed for 30 min with gentle agitation and 5 min in an ultrasound bath, followed by a clarifying spin (20 min, 15,000 g) using a new tube for each step. Human skin equivalents were lysed in RIPA buffer (supplemented with phosphatase and protease inhibitors) [5].

All protein samples were stored at -80 °C. Concentrations were determined prior to the experiment using a Pierce™ 660 nm protein assay kit (Thermo Fisher Scientific, Waltham, MA, USA). All samples were denatured in SDS sample loading buffer (10 %) and electrophoresed on polyacrylamide-gels (7.5 %, SDS-PAGE) in Tris-Glycine buffer (0.1 % SDS). Electroblotting was performed onto polyvinylidene difluoride membranes (PVDF, Immun-Blot®, Bio-Rad) in Tris-Glycine buffer (0.3 % SDS, 20 % methanol, 4 °C). The membranes were blocked in milk (5 %) in Tris-buffered saline supplemented with Tween20 (0.1 vol%) for 45 min.

C) Preparation for immunofluorescence staining

Immunofluorescence staining was performed according to the procedure previously described [1]. *X.* oocytes were fixed in paraformaldehyde (4 %, 3 h) and washed twice with PBS. After dehydration in increasing concentrations of ethanol (2 times in 70 % (1 h), 70 % (overnight), 3 times in 80 % (1 h), 80 % (72 h), 2 times in 96 % (10 min), 3 times in 99.9 % (10 min)) and immersion in Xylene (2 times for

10 min), *X.* oocytes were embedded in paraffin (2 times for 30 min), cut (5 µm) and mounted (Superfrost® Plus Menzel-Gläser, Carl Roth). Subsequently, slides were deparaffinized overnight (Roti®-Histol, Carl Roth) and rehydrated in descending concentrations of ethanol (99.9 %, 96 %, 90 %, 80 %, 70 %, demineralized water, 5 minutes each). After washing (PBS, 5 min) and boiling (EDTA buffer, 1 mmol·L^{-1}; pH 8.0, 15 min), the slides were rinsed in PBS for 1, 5, and 5 minutes respectively.

One hour after seeding HEK-293 cells on coverslips, these were washed twice in PBS, transferred to Roti®-Histofix (4 %, 30 min), and washed again twice in PBS.

Human skin equivalents were submerged in tissue freezing media, flash frozen, and cut into cross sections (8 µm) on a cryotome (Leica Biosystems, PLACE, Wetzlar, Germany) as described previously [5]. Samples were stored at -80 °C. Cryo-embedded human skin equivalents were defrosted at room temperature for 45 min. The tissue was fixated in paraformaldehyde for 5 min and washed 2 times with PBS for 5 min.

X. oocytes, HEK-293 cells, and human skin equivalents were all permeabilized in Triton X-100 (0.5 %, 5 min; Merck KGaA, Darmstadt, Germany) and washed twice in PBS. Afterwards, all preparations were incubated in blocking solution (BS, goat serum (5 %; PAN-Biotech GmbH, Aidenbach, Germany) in PBS) in a closed container for 1 hour. Samples were then stained with primary antibody diluted in BS as stated in methods or in BS only for secondary antibody controls (both 4 °C, overnight). After washing with BS (3 times for 5 min), the slides were incubated with diluted secondary antibodies as stated in methods (in BS supplemented with 4′, 6-diamidine-2′-phenylindole dihydrochloride (1 µg/mL; DAPI, Roche, Mannheim, Germany), 37 °C, 1 h). Afterwards, samples were washed with demineralized water and ethanol, followed by embedding (Mount Flour, Biocyc GmbH & Co. KG, Potsdam, Germany) on a microscopic slide.

D) Double-barrelled pH-sensitive microelectrode measurements

Experiments with pH-sensitive microelectrodes were prepared essentially as described previously [1,7]. For pH-measurements, SUTTER BF 150-86-10 glass tubing (Science Products GmbH, Hofheim, Germany) was used. The reference barrel was made of filamented bisected GC150F 15 glass tubing (Harvard Apparatus, Kent, UK). Two core cable ends (4×10, 611889, Conrad Bauelemente, Conrad Elektronik, Hirschau, Germany) were pushed into tightly fitting shrink tubing (Ø 3 mm) and used to adjoin the two barrels, leaving the middle section free (~ 3 cm). A small piece of shrink tubing (1 mm; Deray-H-set 1/8″, DSG-Canusa, Meckenheim, Germany) was slipped over the end of the reference barrel, slightly separating the barrels at one end. After baking (180 °C, 10 min), the piggyback electrodes were pulled with a programmable multipipette puller (PMP-107, Microdata Instrument, South Plainfield, NJ, USA) to give a resistance of ~ 50 MΩ. Subsequently, the reference barrel was perfused with pressurized dry air via plastic tubing pushed over one end (~ 0.9 bar). The pH-sensitive barrel was pushed into a rubber insert in the lid of a heated glass jar and exposed to the vapour formed by a drop (200 µl) of fresh dichlorodimethylsilane (Sigma-Aldrich) for 30 min. Pipettes were then baked at 180 °C for 2 h. The pH-sensitive barrel was filled with Hydrogen Ionophore I-Cocktail A (0.2 µl; Sigma Aldrich) via a Microliter

syringe (type 7000.50C, Hamilton Company, Reno, NV, USA). The electrodes could be stored in a plastic container with silica gel (P077.2, Carl Roth) and light protection for many months. Once opened, Dichlorodimethylsilane had to be replaced frequently.

On the experimental day, the reference barrel was filled with KCl solution (0.5 mol·L^{-1}) via a MicroFil micropipette (34Gauge/67 mm, World Precision Instruments, Sarasota, FL, USA), while the pH-sensitive barrel was filled with KCl/HEPES-buffer (in mmol·L^{-1}: 500 KCl, 20 HEPES, pH 7.2 adjusted with Tris). Microelectrodes were then trimmed (0.5-30 MΩ) to remove clogging silane residues using a beveller (BV-10, Sutter Instrument, Novato, CA, USA).

Measurements were performed in a continuously perfused bath chamber (23 °C). Via chlorinated silver wires, the two barrels were connected to an amplifier (F-223 A Dual Electrometer, World Precision Instruments). The bath was grounded to a common technical earth via a chlorinated silver wire. In addition, the bath was connected to a commercial electrode (Metrohm, Filderstadt, Germany) via a KCl (3 mol·L^{-1}) agar bridge to minimize liquid junction potential effects (ground signal) [2]. All three signals were measured versus the technical earth and recorded using LabChart 7 software (ADintruments Ltd, Oxford, UK). The potential difference between the two barrels was used to determine the intracellular pH (pH_i), while the potential difference between the reference barrel and the ground signal from the bath corresponded to the membrane potential (U_{mem}). Electrodes were calibrated before and after each measurement using NaCl (pH 7.4) and NaCl-6.4 (pH 6.4) solutions. Suitable electrodes showed a stable potential difference of 45 mV or higher measured by the pH-sensitive barrel with effects at the reference electrode < 0.3 mV. A micromanipulator (Mini 25, Luigs & Neumann, Ratingen, Germany) was used to insert the microelectrode into the *X.* oocyte. Impalement was considered to be successful if a sharp drop in the potential across the reference channel to values under -10 mV could be observed. Drift correction was used to compensate for small variations in the pH-response of the electrode to the calibration solutions before and after the experiment, but cells were excluded when ΔpH was greater than 0.05.

E) Relative permeability ratio

To calculate relative permeability ratios, the assumption was made that leak currents were low and that the contribution of extracellular K^+ and intracellular Cl^- to total membrane potential was low. The Goldman-Hodgkin-Katz (GHK) theory then yields the following expression [6]:

$$(1) \qquad U_\mathrm{A} - U_\mathrm{B} \approx -\frac{\mathrm{R} \cdot \mathrm{T}}{\mathrm{F}} \cdot \ln\left(\frac{\mathrm{P_A} \cdot [\mathrm{A}]_\mathrm{o}}{\mathrm{P_B} \cdot [\mathrm{B}]_\mathrm{o}}\right)$$

Here, U_x designates the membrane potential in solution X, F the Faraday constant, and T the absolute temperature, while P_x designates the permeability, and $[X]_o$ the outside concentration of ion X.

F) Analysis of Inside-out patch-clamp data via Goldman-Hodgkin-Katz theory

Igor (Igor Pro 6.2.2.2; WaveMetrics Inc., Lake Oswego, USA) was used to fit amplitude histograms to a Gaussian distribution as described previously [4,10,7]. The unitary current was determined from the

distance of the maxima and cross-checked by direct measurements. Current-Voltage plots (IV-plots) were obtained by plotting the obtained unitary currents against the clamped pipette potentials, which were corrected for liquid junction potentials throughout (JPCalcWin software, School of Medical Sciences, Sydney, Australia) [2]. The GHK theory was used to fit the IV-plot via Igor, yielding the permeability for two ions (P_A and P_B) as described in detail in [10].

$$(2)\quad I = \frac{U_{mem} \cdot F^2}{R \cdot T} \cdot \left(\frac{P_A \cdot [A]_i + P_B \cdot [B]_i - (P_A \cdot [A]_o + (P_B \cdot [B]_o) \cdot e^{-U_{mem} \cdot \frac{F}{R \cdot T}}}{1 - e^{-U_{mem} \cdot \frac{F}{R \cdot T}}} \right)$$

If pipette and bath solutions were identical (symmetrical configuration), the slope of the linear regression of the IV-plot was used to determine the conductance (G_x) of a given ion X. Equation (3) was used to yield P_x, the permeability of the ion X, while [C] is the concentration of the major cation in used solutions (96 mmol·L^{-1}).

$$(3)\quad G_X = \frac{I}{U_{mem}} = \frac{F^2}{R \cdot T} \cdot P_X \cdot [C]$$

To compare these conductance values with data obtained with HEK-293 cells, where mammalian Ringer solutions containing 145 mmol·L^{-1} of the main cation, the conductance G_x in oocyte solution was multiplied by the concentration ratio yielding:

$$(4)\quad G_X\,(145\ \text{mmol}\cdot\text{L}^{-1}) = G_X\,(96\ \text{mmol}\cdot\text{L}^{-1}) \times \frac{145\ \text{mmol}\cdot\text{L}^{-1}}{96\ \text{mmol}\cdot\text{L}^{-1}}$$

For comparison of single-channel data from overexpressing cells and controls, data were plotted in amplitude histograms (Fig. 9 and Fig. S3). After collecting data from all patches, the total conductance range of all measurements was divided into a number of equidistant bins, which were plotted on the horizontal axis. The vertical axis was used to plot the number of patches with a conductance falling into the corresponding bin on the X-axis. Accordingly, the bars in these histograms do not correspond to discrete conductance steps.

G) Attempts to enhance cell viability of HEK-293 cells overexpressing G573S

In order to enhance the viability of G573S HEK-293 cells for patch-clamping, we attempted various approaches to reduce the concentration of free Ca^{2+} by diluting the medium (DMEM, FG 0445; Ca^{2+}-concentration: 200 mg·L^{-1} = 0.91 mmol·L^{-1}) with PBS (no Ca^{2+} or Mg^{2+}; Sigma-Aldrich) or by supplementing EGTA in PBS (10 mmol·L^{-1}). Furthermore, we tried out different concentrations of foetal bovine serum (FBS, Biochrom). The different strategies are summarised in Table S1 but no approach yielded the desired outcome of cells emitting a visible fluorescent signal allowing selection for patch-clamping.

Additionally, the duration for transfection with the vector was altered. We transfected HEK-293 cells with the G573S mutant and control vector for 22 h, 93 h, 2 h, and 1 h, respectively, but no fluorescent signal emerged despite successful parallel expression of wild type hTRPV3. Most likely, the cells started to disintegrate immediately after expression of the G573S mutant but prior to the downstream expression of observable quantities of GFP.

This hypothesis was confirmed via subsequent immunofluorescence. A few cells could be shown to express the G573S mutant with traces of visible GFP fluorescence. In addition, disintegrated cells could be seen (see Fig. 14a; 22 h transfection). It is likely that the majority of cells successfully expressing the G573S mutant rapidly became apoptotic and detached from the coverslip before the downstream expression of GFP could occur. It was therefore not possible to select successfully expressing cells for patch-clamping.

#	Medium [mL]	$PBS^{-/-}$ [mL]	EGTA [$mmol\cdot L^{-1}$]	FBS [µL]	Free Ca^{2+} [$mmol\cdot L^{-1}$]
1	2.5	2.5	0	250	0.5
2	3.75	1.25	0	125	0.7
3	4.5	0.5	0	50	0.8
4	1	1	0	100	0.5
5	1.5	0.5	0	50	0.7
6	1.8	0.2	0	20	0.8
7	1	0	0	0	0.9
8	1	0	0.2	4	0.7
9	1	0	0.4	0	0.5
10	1	0	0.6	8	0.3
11	1	0	0.8	10	0.07
12	1	0	1.0	20	0.006
13	1	0	5.0	100	0.0001

Table S1. Composition of different media to enhance the vitality of for G573S HEK-293 cells

H) Supplemental microelectrode data: hTRPV3, bTRPV3, and control *X.* oocytes

The double-barrelled pH-sensitive microelectrode experiments (Fig. 5 and Fig. S1) were performed in parallel to our study of the bovine homologue of TRPV3 (bTRPV3), alternating between hTRPV3, bTRPV3, and control *X.* oocytes [7].

Screening experiments

In preliminary screening experiments on four hTRPV3 overexpressing *X.* oocytes, Na^+ in the bath solution was consecutively replaced by K^+, NH_4^+, or $NMDG^+$ while Cl^- was replaced by Glu^- (Fig. S1).

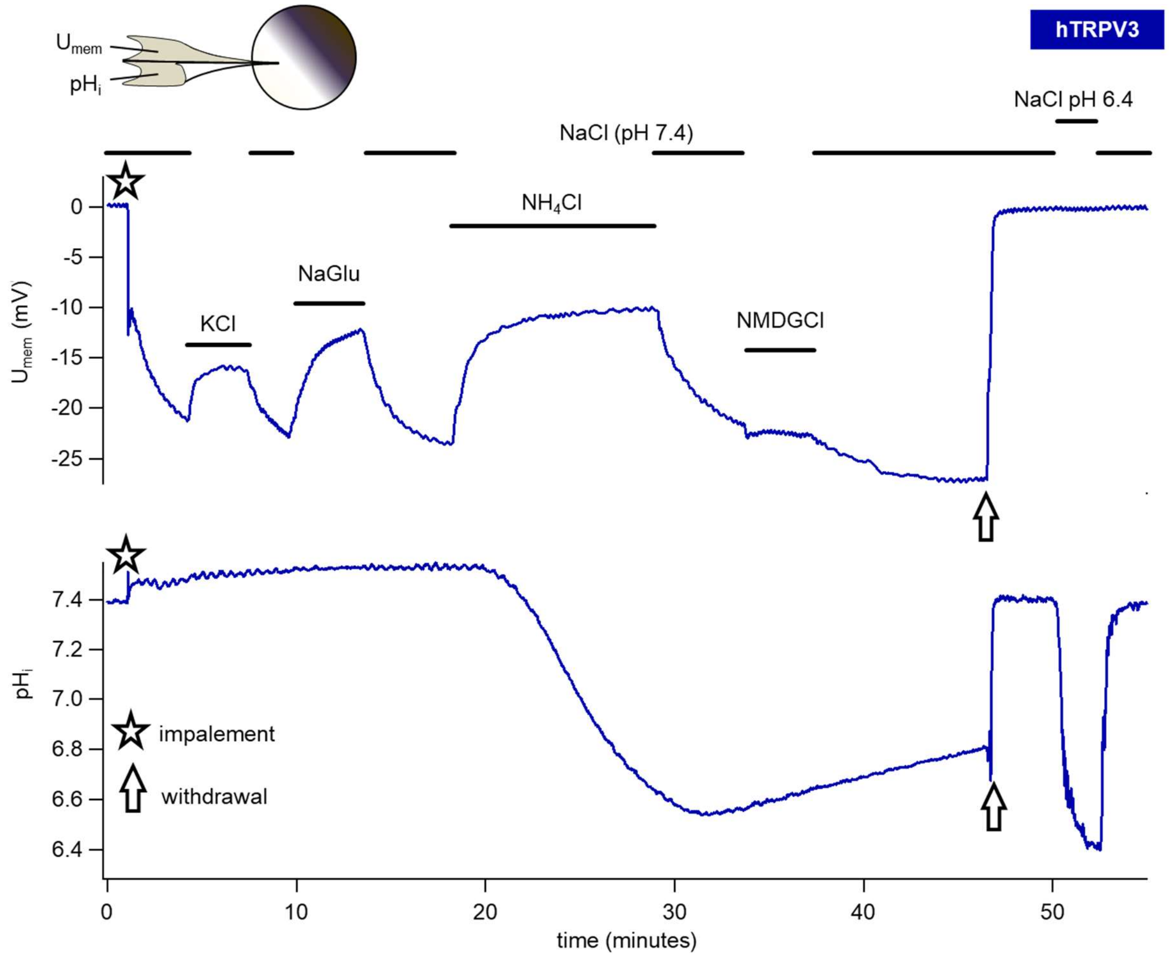

Fig. S1 Double-barrelled pH-sensitive microelectrodes: original recording showing the response of an hTRPV3 *X.* oocyte to various solutions

At the beginning of the measurement, impalement (star) can be seen, which resulted in a sharp drop in U_{mem} with a small rise in pH_i. Opposite effects visible after withdrawal of the pipette (arrow) at the end. Replacement of Na^+ by K^+ or Cl^- by Glu^- led to a reversible depolarisation of U_{mem} with no impact on pH_i. Conversely, NH_4^+ led to both a rapid depolarisation of U_{mem} and a slower decrease in pH_i, arguing for influx of NH_4^+. In line with this, return to NaCl solution led to a rapid repolarization of U_{mem} although recovery of pH_i was very slow (see discussion). Replacement of Na^+ by $NMDG^+$ had no effect on the recovery of pH_i, arguing against an involvement of NHE in pH regulation. Effects of $NMDG^+$ on U_{mem} were variable

X. oocytes (n/N = 4/1) depolarised after application of KCl (-12.1 ± 1.8 mV), NaGlu (-7.8 ± 2.0 mV), or NH_4Cl (-4.9 ± 1.9 mV) with recovery after each return to NaCl solution (-17 ± 3 mV). However, only the depolarisation in NaGlu (p = 0.03) and NH_4Cl (p = 0.006) tested for significance. From the reversal

potentials, relative permeability ratios could be calculated ([6] and supplement part E), yielding a $p(Cl^-)/p(Glu^-)$ of 1.62 ± 0.25. Permeability to NH_4^+ ($p(NH_4^+)/p(Na^+)$ = 1.79 ± 0.22) was significantly higher than that to K^+ ($p(K^+)/p(Na^+)$ = 1.33 ± 0.12, p = 0.03).

In NaCl, pH_i was 7.40 ± 0.05 and solution changes to KCl (p = 0.9) or NaGlu (p = 0.8) showed no significant effect. Application of NH_4Cl solution resulted in a significant acidification (6.64 ± 0.09 after 10 minutes, p ≤ 0.001) with a slow recovery after return to NaCl solution (6.72 ± 0.08 after 15 minutes). This recovery was not interrupted by removal of Na^+, so that any involvement of NHE seems unlikely. In response to $NMDG^+$, two cells showed hyperpolarisation (ΔU_{mem} = -3.8 ± 0.5 mV), in line with a reduction of Na^+ influx. Surprisingly, two other cells depolarised (ΔU_{mem} = 2.4 ± 1.7 mV). Similar effects have been observed by others and possibly reflect membrane leakage induced by full substitution of Na^+ by $NMDG^+$ [7,8]. Such high concentrations of $NMDG^+$ can induce pore dilation of TRP channels with influx of large molecular substrates [10,3]. It is possible to speculate that this leads to uptake of $NMDG^+$, swelling and bursting of cells, but further work is clearly necessary. For the preliminary experiments, observations were similar to those seen in bTRPV3 and control oocytes [7].

Comparison between bRPV3, hTRPV3, and control oocytes

The acidification by NH_4Cl could be confirmed in more rigorously performed experiments. Membrane potentials, intracellular pH (pH_i), and relative permeabilities are visualised in Fig. S2 and statistically evaluated in Table S2. The relative permeability ratio of Na^+ versus $NMDG^+$ was comparable between hTRPV3 and bTRPV3 (p = 0.5), but significantly higher than that of the controls (p = 0.002 ctrl vs bTRPV3 [7] and p = 0.005 vs hTRPV3 (main text)), reflecting expression of TRPV3. After application of NH_4Cl solution, all *X.* oocytes depolarised strongly and acidified, reflecting expression of NH_4^+ conducting channels by all three groups. However, after 3.5 minutes of incubation with NH_4Cl, both hTRPV3 and bTRPV3 *X.* oocytes showed a lower value of pH_i than controls (p = 0.004 and p = 0.04 [7], respectively), reflecting a higher influx rate of NH_4^+. The pH_i dropped most rapidly in hTRPV3, showing a slight recovery towards the end of the measurement which suggests that an equilibrium distribution had been reached in this group of oocytes. This may explain why a removal of Ca^{2+} had no further effect on pH_i in this group. However, both hTRPV3 and bTRPV3 oocytes depolarised after removal of Ca^{2+} as to be expected after opening of a divalent-sensitive cation channel such as TRPV3. Conversely, control cells hyperpolarised, possibly reflecting expression of Ca^{2+} inactivated chloride channels [12,9]. For further discussion of the differences between hTRPV3 and bTRPV3, see the main text and supplement part I, Fig. S4.

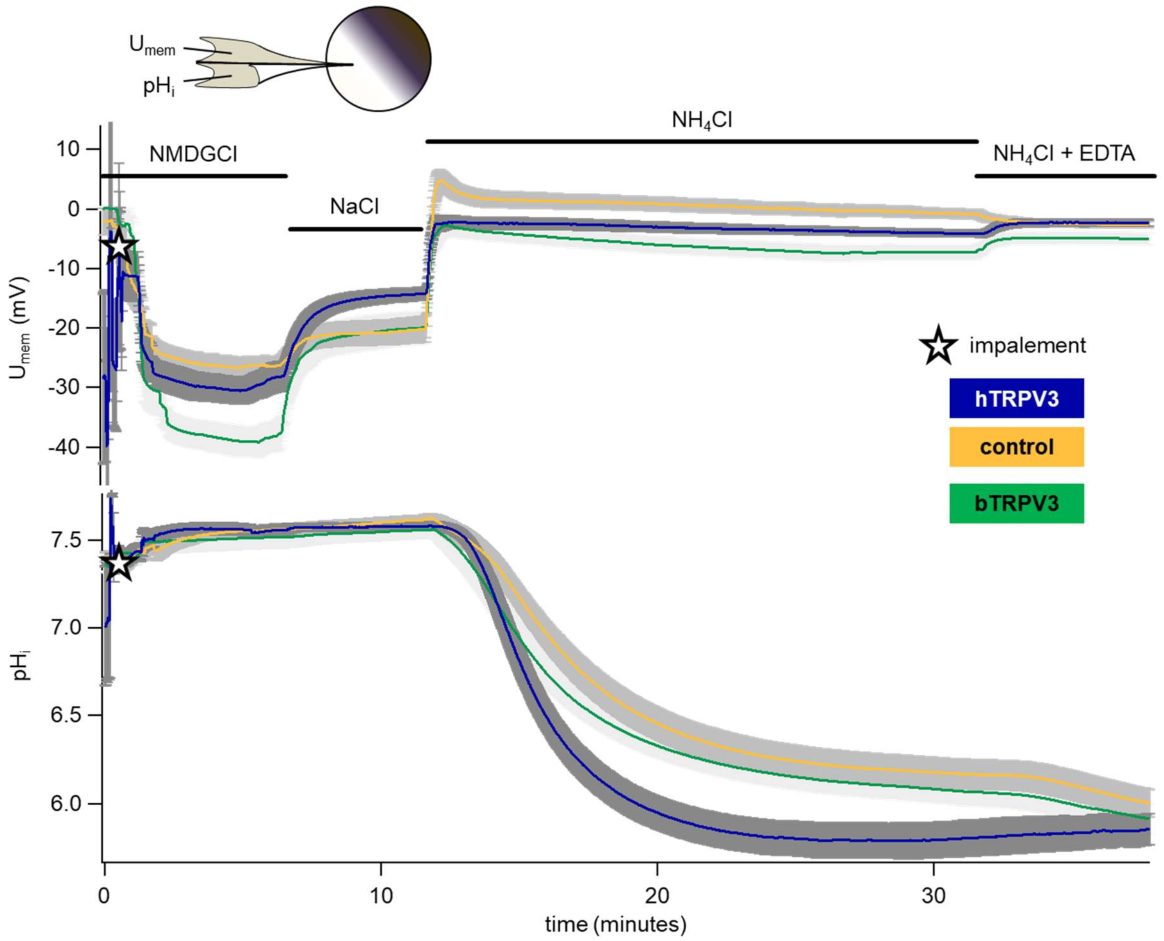

Fig. S2 Double-barrelled pH-sensitive microelectrode measurement of *X.* oocytes

Shown are the mean of the membrane potential and pH_i of X. oocytes expressing hTRPV3 (n/N = 12/3, blue), bTRPV3 (n/N = 14/3, green) [7], and controls (n/N = 16/3, orange). The SEM is visualised in different shades of grey. The differences between hTRPV3 and bTRPV3 most likely reflect a higher expression efficiency in hTRPV3, as discussed in supplement part **I**, Fig. S4

**Membrane potential [mV]**							
Bath solution	hTRPV3	control	bTRPV3	ANOVA	bV3 vs ctrl	hV3 vs ctrl	hV3 vs bV3
NMDGCl	-30.6 ± 2.2[a]	-27.2 ± 1.8[a]	-39.2 ± 2.6[a]	0.003	0.001	0.4	0.02
NaCl	-14.4 ± 1.1[b]	-20.3 ± 2.2[b]	-20.1 ± 2.8[b]	0.02	0.6	0.03	0.009
NH_4Cl (3.5 min)	-2.5 ± 1.0[c]	1.5 ± 1.4[c]	-4.4 ± 1.5[c]	0.005	0.003	0.03	0.3
NH_4Cl (20 min)	-4.1 ± 0.6[d]	-0.9 ± 0.8[d]	-7.2 ± 1.3[d]	<0.001	<0.001	0.003	0.2
NH_4Cl-EDTA	-2.3 ± 0.6[c]	-2.7 ± 0.5[e]	-5.0 ± 1.1[c]	0.03	0.03	0.7	0.02
**Intracellular pH (pH_i)**							
	hTRPV3	control	bTRPV3	ANOVA	bV3 vs ctrl	hV3 vs ctrl	hV3 vs bV3
NMDGCl	7.56 ± 0.04[a]	7.56 ± 0.04[a]	7.52 ± 0.07[a]	1.0	1.0	0.9	0.9
NaCl	7.58 ± 0.03[a]	7.61 ± 0.03[b]	7.56 ± 0.05[b]	0.8	0.9	0.5	0.8
NH_4Cl (3.5 min)	6.76 ± 0.09[b]	7.14 ± 0.06[c]	6.90 ± 0.08[c]	< 0.001	0.04	0.004	0.3
NH_4Cl (20 min)	5.81 ± 0.11[c]	6.18 ± 0.09[d]	6.08 ± 0.08[d]	0.02	0.6	0.007	0.03
NH_4Cl-EDTA	5.85 ± 0.09[c]	6.05 ± 0.08[e]	5.96 ± 0.09[e]	0.2	0.6	0.07	0.3
**Change of pH_i (slope in ΔpH/min)**							
	hTRPV3	control	bTRPV3	ANOVA	bV3 vs ctrl	hV3 vs ctrl	hV3 vs bV3
NMDGCl	0.03 ± 0.02[a]	0.01 ± 0.01[a]	-0.02 ± 0.02[a]	0.7	0.7	0.5	0.6
NaCl	0.00 ± 0.00[a]	0.00 ± 0.01[a]	0.03 ± 0.01[b]	0.009	0.02	0.6	0.003
NH_4Cl (3.5 min)	-0.39 ± 0.04[b]	-0.22 ± 0.04 [b]	-0.22 ± 0.03[c]	0.004	0.7	0.006	0.002
NH_4Cl (20 min)	0.02 ± 0.01[a]	0.00 ± 0.01[a]	-0.01 ± 0.01[a]	0.13	0.9	0.05	0.2
NH_4Cl-EDTA	-0.004± 0.02[a]	-0.04 ± 0.02 [c]	-0.02 ± 0.01[a]	0.02	0.3	0.01	0.05
**Relative permeability ratio p(X) / p($NMDG^+$)**							
Ion X	hTRPV3	control	bTRPV3	ANOVA	bV3 vs ctrl	hV3 vs ctrl	hV3 vs bV3
Na^+	1.98 ± 0.18[a]	1.37 ± 0.12[a]	2.25 ± 0.20[a]	0.002	0.002	0.005	0.5
NH_4^+ (20 min)	2.93 ± 0.24[b]	2.93 ± 0.23[b]	3.62 ± 0.25[b]	0.08	0.06	1.0	0.05
NH_4^+ (EDTA)	3.15 ± 0.25[c]	2.70 ± 0.18[c]	4.01 ± 0.33[c]	0.008	0.003	0.3	0.07

Table S2. Double-barrelled pH-sensitive microelectrodes: Effects of NH_4^+ on the pH_i and the membrane potential of *Xenopus* oocytes expressing hTRPV3 (hV3, n/N = 13/3), bTRPV3 (bV3, n/N = 14/3) [7] and controls (ctrl; n/N = 16/3)

Solutions were applied consecutively and values were measured 5 minutes after exposure unless indicated otherwise. Within columns, different superscripts indicate significant differences with $p \leq 0.05$. The fifth column gives the p-value of the ANOVA (Kruskal-Wallis) between the three groups, followed by the p-values for pairwise comparisons (Mann-Whitney) in column 6, 7, and 8

I) Comparison of single-channel patch-clamp data: hTRPV3, bTRPV3, and control *X.* oocytes

Single-channel patch-clamp experiments of *X.* oocytes overexpressing hTRPV3 were performed in parallel to our study of bTRPV3 [7], again allowing a direct comparison. The resulting histograms are shown in Fig. S3 and discussed in context with the microelectrode results in Fig. S4.

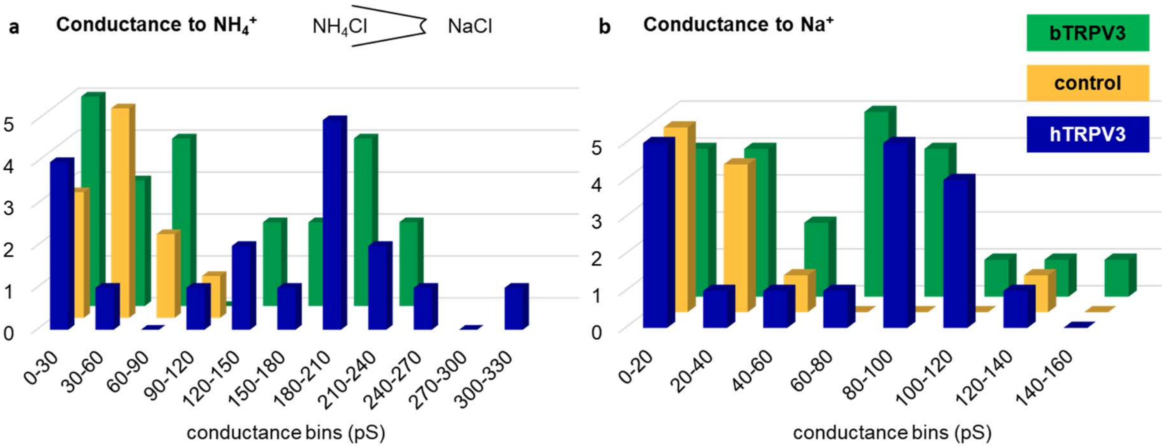

Fig. S3. Histograms of single-channel conductances from *X.* oocytes overexpressing hTRPV3, bTRPV3 [7], and controls

a) Histogram of all single-channel conductances for NH_4^+, determined from GHK fits of IV-plots of experiments in the asymmetrical configuration with NH_4Cl in the pipette and NaCl in the bath. The vertical axis shows the number of patches (n) falling into the corresponding conductance range or bin shown on the horizontal axis. Patches from control *X.* oocytes only showed NH_4^+ conductances below 100 pS. In contrast, *X.* oocytes expressing hTRPV3 or bTRPV3 (published in Liebe et al. [7]) showed both small channels such as those observed in the control *X.* oocytes and larger conductances ranging up to 303 pS. These larger conductances probably reflected heteromers of endogenous channels with TRPV3. b) Corresponding histogram of all single-channel conductances for Na^+. A similar pattern emerged as in a), with a cluster of channels below 60 pS in patches from both control and TRPV3 *X.* oocytes, and a second, larger cluster emerging in hTRPV3 and bTRPV3 *X.* oocytes. Interestingly, one solitary control patch expressed a very large conductance to Na^+ reflecting what is clearly a very diverse population of channels expressed by the native *X.* oocytes [12]. Any differences between bTRPV3 and hTRPV3 did not test for significance

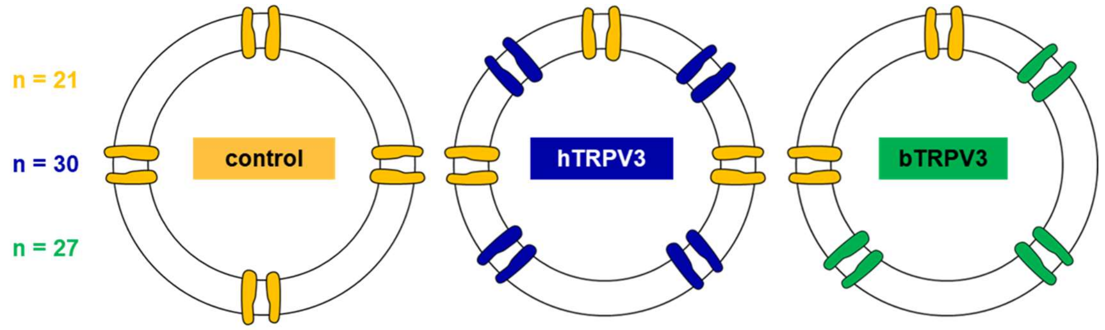

	control	hTRPV3	bTRPV3
no channel activity	7/21 (33%)	6/30 (20%)	4/27 (15%)
small conductance (<100 pS)	14/21 (67%)	16/30 (53%)	9/27 (33%)
large conductance (>100 pS)	0/21 (0%)	19/30 (63%)	14/27 (52%)

Fig. S4. Schematic representation showing the relative abundance of small and large conductances in single-channel patch-clamp experiments for hTRPV3, bTRPV3 [7], and control *X.* oocytes in symmetrical NH_4Cl solution

Small channels (<100 pS) were primarily expressed by control *X.* oocytes and are shown in yellow. Large channels (>100 pS) are shown in blue if expressed by hTRPV3 oocytes and green if expressed by bTRPV3 oocytes. The number n of patches with either no single-channel events, small channels (<100 pS), or large channels (>100 pS) is given in relation to the total number N of patches measured as a ratio (n/N) followed by the percentage. 15 hTRPV3 and 8 bTRPV3 membrane patches simultaneously expressed both small and large conductances. The hTRPV3 *X.* oocytes showed both a higher number of channels in individual patches and a larger percentage of large channels than bTRPV3 *X.* oocytes.

We suggest that expression of hTRPV3 or bTRPV3 significantly enhanced the single-channel conductance of individual channels, with the scatter in values reflecting the formation of heteromers with endogenous channels. Furthermore, expression of endogenous channels was suppressed by either hTRPV3 or bTRPV3, with effects more pronounced in the case of bTRPV3. It also appears that expression of hTRPV3 was more efficient than expression of bTRPV3. Both the lower number of bTRPV3 channels and the lower number of endogenous channels might explain why bTRPV3 oocytes did not acidify as rapidly in response to NH_4Cl as hTRPV3 oocytes in the microelectrode experiments (supplement part H).

References

1. Abdoun K, Stumpff F, Rabbani I, Martens H (2010) Modulation of urea transport across sheep rumen epithelium in vitro by SCFA and CO2. American journal of physiology Gastrointestinal and liver physiology 298:G190-202. doi:10.1152/ajpgi.00216.2009
2. Barry PH, Lynch JW (1991) Liquid junction potentials and small cell effects in patch-clamp analysis. J Membr Biol 121:101-117. doi:10.1007/BF01870526
3. Ferreira LG, Faria RX (2016) TRPing on the pore phenomenon: what do we know about transient receptor potential ion channel-related pore dilation up to now? J Bioenerg Biomembr 48:1-12. doi:10.1007/s10863-015-9634-8
4. Georgi MI, Rosendahl J, Ernst F, Gunzel D, Aschenbach JR, Martens H, Stumpff F (2014) Epithelia of the ovine and bovine forestomach express basolateral maxi-anion channels permeable to the anions of short-chain fatty acids. Pflugers Arch 466:1689-1712. doi:10.1007/s00424-013-1386-x
5. Giulbudagian M, Yealland G, Hönzke S, Edlich A, Geisendörfer B, Kleuser B, Hedtrich S, Calderón M (2018) Breaking the Barrier - Potent Anti-Inflammatory Activity following Efficient Topical Delivery of Etanercept using Thermoresponsive Nanogels. Theranostics 8:450-463. doi:10.7150/thno.21668
6. Hille B (2001) Ion Channels of Excitable Membranes. 3rd edn. Sinauer Associates, Sunderland, Mass. doi:10.4236/jbm.2020.82005
7. Liebe F, Liebe H, Kaessmeyer S, Sponder G, Stumpff F (2020) The TRPV3 channel of the bovine rumen: localization and functional characterization of a protein relevant for ruminal ammonia transport. Pflügers Archiv - European Journal of Physiology 472:693-710. doi:10.1007/s00424-020-02393-2
8. Ochoa-de la Paz LD, Espino-Saldana AE, Arellano-Ostoa R, Reyes JP, Miledi R, Martinez-Torres A (2013) Characterization of an outward rectifying chloride current of Xenopus tropicalis oocytes. Biochim Biophys Acta 1828:1743-1753. doi:10.1016/j.bbamem.2013.03.013
9. Reifarth FW, Amasheh S, Clauss W, Weber W (1997) The Ca2+-inactivated Cl- channel at work: selectivity, blocker kinetics and transport visualization. J Membr Biol 155:95-104. doi:10.1007/s002329900161
10. Schrapers KT, Sponder G, Liebe F, Liebe H, Stumpff F (2018) The bovine TRPV3 as a pathway for the uptake of Na+, Ca2+, and NH4+. PLoS One 13:e0193519. doi:10.1371/journal.pone.0193519
11. Vitzthum C, Stein L, Brunner N, Knittel R, Fallier-Becker P, Amasheh S (2019) Xenopus oocytes as a heterologous expression system for analysis of tight junction proteins. Federation of American Societies for Experimental Biology Journal 33:5312-5319. doi:10.1096/fj.201801451RR
12. Weber W (1999) Ion currents of Xenopus laevis oocytes: state of the art. Biochim Biophys Acta 1421:213-233. doi:10.1016/s0005-2736(99)00135-2
